# Explicit Consideration of Temperature Improves Predictions of Toxicokinetic–Toxicodynamic Models for Flupyradifurone and Imidacloprid in *Gammarus pulex*

**DOI:** 10.1021/acs.est.2c04085

**Published:** 2022-10-25

**Authors:** Annika Mangold-Döring, Anna Huang, Egbert H. van Nes, Andreas Focks, Paul J. van den Brink

**Affiliations:** † Department of Aquatic Ecology and Water Quality Management, Wageningen University and Research, P.O. Box 47, 6700 AAWageningen, The Netherlands; ‡ System Science Group/Institute of Mathematics, Osnabrück University, Barbarastr. 12, D-49076Osnabrück, Germany; § Wageningen Environmental Research, P.O. Box 47, 6700 AAWageningen, The Netherlands

**Keywords:** TK−TD, GUTS, effect modeling

## Abstract

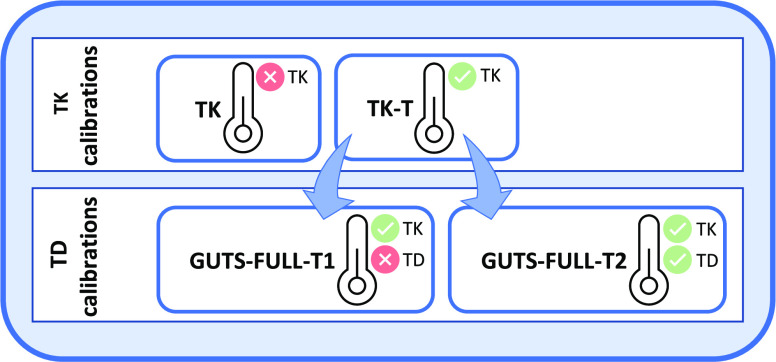

In the face of global climate change, where temperature fluctuations and the frequency of extreme weather events are increasing, it is needed to evaluate the impact of temperature on the ecological risk assessment of chemicals. Current state-of-the-art mechanistic effect models, such as toxicokinetic–toxicodynamic (TK–TD) models, often do not explicitly consider temperature as a modulating factor. This study implemented the effect of temperature in a widely used modeling framework, the General Unified Threshold model for Survival (GUTS). We tested the model using data from toxicokinetic and toxicity experiments with *Gammarus pulex* exposed to the insecticides imidacloprid and flupyradifurone. The experiments revealed increased TK rates with increasing temperature and increased toxicity under chronic exposures. Using the widely used Arrhenius equation, we could include the temperature influence into the modeling. By further testing of different model approaches, differences in the temperature scaling of TK and TD model parameters could be identified, urging further investigations of the underlying mechanisms. Finally, our results show that predictions of TK–TD models improve if we include the toxicity modulating effect of temperature explicitly.

## Abstract 2

The underlying physiological mechanisms of temperature effect on the toxicity of pesticides are poorly understood. This study indicates that applying mechanistic effect models to experimental data gains insights beyond the standard toxicity information to address this knowledge gap.

## Introduction

As an environmental factor whose variability is intensifying in the course of global climate change, temperature is expected to play an increasing role in the environmental fate and toxic effects of contaminants.^1−4^ However, temperature variability is not included in standard toxicity tests of contaminants, which are usually conducted at one constant temperature (e.g., OECD Test No. 211).^5^ Fortunately, an increasing number of studies include the role of temperature on the effect of contaminant toxicity. The majority of such studies using aquatic organisms showed increased toxicity with increasing temperature (reviewed in Heugens et al.,^6^ Holmstrup et al.,^7^ and Noyes et al.^3^). Nevertheless, also decreased toxicity with increasing temperature has been observed, for instance, for DDT (dichlorodiphenyltrichloroethane) and pyrethroid insecticides.^8^ These contradictory results underline the demand to explicitly consider the impact of temperature in studies for environmental risk assessments (ERAs), aiming to reduce uncertainty.

Mechanistic or process-based models, such as toxicokinetic–toxicodynamic (TK–TD) models, are a powerful tool to investigate contaminant’s effects and allow screening of many exposure scenarios. In combination with experiments, previous TK–TD model applications have been proven useful to test hypotheses on temperature-modified toxicity and its underlying mechanisms. For instance, Heugens et al.^9^ showed in a model study combined with experiments that the temperature effect on cadmium toxicity of *Daphnia magna* could not be ascribed to accumulation kinetics alone; the altered susceptibility of the daphnids also played a vital role. Nonetheless, how temperature modulates both TK and TD processes is rarely studied. However, there have been proposals (see the Methods section) on how to account for temperature^10^ in current state-of-the-art effect modeling approaches, e.g., in the widely used General Unified Threshold model of Survival (GUTS).^11^

GUTS is a TK–TD model framework deemed ready for use in ERA by the European Food Safety Authority.^12^ Recently, Gergs et al.^13^ included the effect of temperature in a simplified GUTS model to illustrate the influence of temperature on the sensitivity of many species. For their study, they used the reduced GUTS version (GUTS-RED), which does not allow to separately investigate the temperature influence on TK and TD model parameters. To the best of our knowledge, a systematic evaluation of the temperature scaling for both TK and TD parameters has not been done for the GUTS-FULL model.

**Figure 1:**
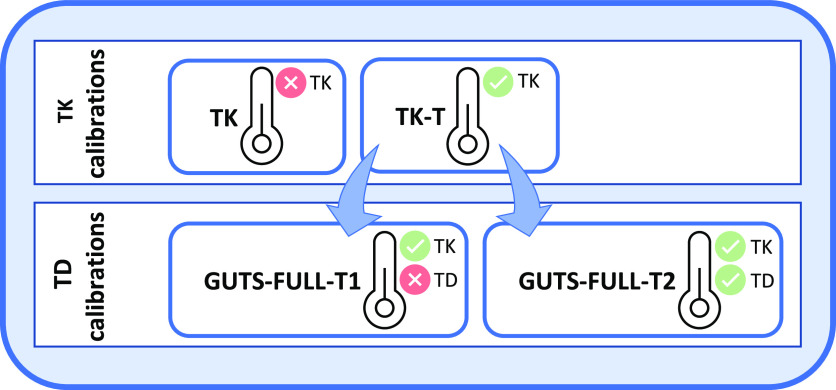
Overview of the used toxicokinetic (TK) and toxicodynamic (TD) calibration approaches. **TK calibrations** (data: measured internal concentrations over time) TK: Data sets from different temperatures are used as replicates, no consideration of temperature. Assumption: TK is not influenced by temperature. TK-T: Data sets from different temperatures are used simultaneously, explicit consideration of temperature by applying the Arrhenius equation. Assumption: TK is influenced by temperature and can be captured by the Arrhenius equation. **TD calibrations** (data: measured survival over time) GUTS-FULL-T1: data sets from different temperatures are used simultaneously; TK-T parameters are used and corrected for temperature by the Arrhenius equation. Assumption: While TK is influenced by temperature, TD is not influenced by temperature. GUTS-FULL-T2: Data sets from different temperatures are used simultaneously; TK-T parameters are used and corrected for temperature by the Arrhenius equation along with TD parameters. Assumption: Both TK and TD are influenced by temperature.

This research aims to understand the influence of temperature on TK–TD processes through GUTS modeling, applied to toxicity data of the freshwater arthropod *G. pulex* exposed to the insecticides imidacloprid (IMI) and flupyradifurone (FPF). For this, we used the results of previously published toxicity experiments.^14^ To account for temperature effects on the TK processes, we applied the widely used Arrhenius equation^15^ to correct TK rates for different exposure temperatures (Figure 1, TK calibrations). Further, we investigate if only correcting the TK parameter in a GUTS application is sufficient to account for changes in toxicity at different temperatures or if TD model parameters need to be corrected additionally (Figure 1, TD calibrations).

## Materials and Methods

### Toxicokinetics (TK) and Toxicodynamics (TD) in the GUTS Framework

GUTS simulates the probability of death of individuals over time, based on TD processes in connection to a simulated internal concentration of an insecticide resulting from its TK.^10^ We applied the full version of the model (GUTS-FULL), which is calibrated on both survival data and internal concentrations. These data were obtained from TK and toxicity experiments previously published.^14^

Starting from scripts available within the Bring Your Own Model (BYOM) modeling platform (www.debtox.info/byom.html, version 6.2), we extended the TK model and GUTS model with a temperature correction. The values for the model parameters (Supporting Information 01, Table S1) were optimized based on the parameter space explorer.^16^ With samples of the parameter space explorer, the confidence intervals of model curves were produced. All calculations were performed in MATLAB 2021b. MATLAB scripts can be downloaded from GitHub (https://github.com/NikaGoldring/Temperature-explicit-TK-TD).

The TK rates are derived from first-order one-compartment models. With this, we represent the organism as one well-mixed compartment. Insecticides are taken up to and are eliminated from this compartment following first-order kinetics. Only limited knowledge of the biotransformation products of the insecticides was obtained. For FPF, no biotransformation products were measured at any temperature; thus, the TK of FPF was described by a simple first-order bioconcentration model (Supporting Information 01, Table S1 and eq S2a). For IMI, the metabolite IMI-olefin was only detected in the experiments conducted at 18 and 24 °C after 72 and 48 h, respectively. Considering the biotransformation of IMI into imidacloprid-olefin (IMI-ole), these results were calibrated to a TK model with first-order metabolism (Supporting Information 01, Table S1 and eqs S2b and S2c).

During the TK model calibrations, we also calculated the kinetic bioconcentration factor (BCF_kin_)^17^ for FPF (eq 1) and IMI (eq 2) based on the calibrated TK rates. For the CIs of the BCF_kin_ values, a sample from the parameter space explorer was used.^16^
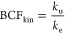
1
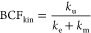
2

The TD processes are based on internal damage *D*_i_ (Supporting Information 01, Table S1, eq S3), which can accumulate in the organism and from which organisms can recover. Model assumptions concerning the mechanism that links this damage to the death of the organism can be either the stochastic death (SD; Supporting Information 01, Table S1 and eqs S4 and S5) or the individual tolerance (IT; Supporting Information 01, Table S1 and eqs S6–S9) approach.^10^

We performed different calibration approaches testing different assumptions on the influence of temperature on the model parameters (Figure 1). To account for the influence of (experimental) temperature on model parameters, we used the Arrhenius equation^10,15,18^ (Supporting Information 01, Table S1 and eq S1). The Arrhenius temperature *T*_A_ was estimated as an additional parameter along with standard GUTS model parameters. We added two such parameters, *T*_A_-tk and*T*_A_-td, to allow the investigation of temperature influence on the TK and TD parts separately. The resulting models were calibrated to data from the previously published TK experiments (conducted at 7, 18, and 24 °C) and toxicity experiments (conducted at 7, 11, and 15 °C) with *G. pulex* exposed to IMI and FPF.^14^ Briefly, both experiments were conducted with field caught *G. pulex* from the Heelsumse brook (coordinates 51.973400, 5.748697). During the 5-day period of the TK experiments, the organisms were not fed, and their body size was 6.87 mm, sd: 0.96 mm. Slightly smaller organisms (i.e., 5.23 mm, sd: 1.09 mm) were used during the 28-day chronic exposure, during which organisms were fed with *Populus* leaves.

### TK Calibrations

Measured internal concentrations of FPF, IMI, and IMI-ole in *G. pulex* were used to calibrate a first-order one-compartment TK model. To evaluate if and how temperature influences the uptake, biotransformation, and elimination of the insecticides, different model calibrations (Figure 1, TK calibrations) were done (per insecticide):

(i) for all temperature data sets simultaneously without temperature correction (TK) and
(ii) for all temperature data sets simultaneously with temperature correction (TK-T), explicitly considering temperature.

For the TK-T model, we extended the TK models with the Arrhenius equation to correct the rates *k_x_* (i.e., uptake rate *k*_u_, elimination rate *k*_e_, formation rate *k*_m_, and elimination rate of the metabolite *k*_em_) for the respective experimental temperatures *T*, using eq 3
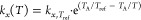
3with the Arrhenius temperature *T*_A_ and the reference temperature *T*_ref_ (here, 20 °C = 293.15 K). The respective TK-T model for FPF was
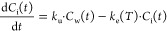
4with *C*_w_ as the exposure concentration (i.e., concentration in the water) and *C*_i_ as the internal concentration. Note, *k*_u_ was not corrected for temperature (see the Results section).

And the TK-T model for IMI was
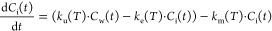
5
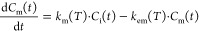
6with *C*_m_ as the concentration of the metabolite IMI-ole.

### TD Calibrations

The survival data of *G. pulex* in all three tested temperatures were used to calibrate the two options of the GUTS model (i.e., GUTS-FULL-SD and GUTS-FULL-IT). Internal concentrations have not been measured in the chronic survival experiments over time. Thus, the previously calibrated TK-T model parameters were fixed in the GUTS-FULL model calibrations (Figure 1, TD calibrations).

Model calibration was done in two versions that follow different assumptions: The first version assumes that it is sufficient to correct only the TK parameters (i.e., as in TK-T) to capture the survival probability at different temperatures. In this version, hereafter referred to as GUTS-FULL-T1, TD parameters were calibrated without temperature correction. In the second version, hereafter referred to as GUTS-FULL-T2, besides the TK parameters, also the TD parameters with time in their dimension were corrected with the Arrhenius equation to evaluate the temperature effect (Figure 1, TD calibrations). For these GUTS-FULL-T2 models, eqs S3, S4, and S9 were adapted as follows
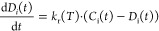
7
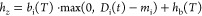
8
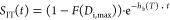
9

Please refer to Supporting Information 01, Table S1 for the parameter explanations.

### Model Evaluation and Toxicity Predictions

Finally, we compared the model fits using Akaike’s information criterion (AIC). Model fits of the same data set were assumed to be substantially different if the difference in their AIC values was >10, (according to Burnham and Anderson^19^) while the model fit with the smallest AIC value was deemed the best one. Additionally, a visual examination was conducted for the interpretation of the results. With the best-fitting parameter set, we then predicted LC_10_ and LC_50_ values for both insecticides at different temperatures (i.e., 7, 11, 15, 18, 20, 24 °C) with their 95% confidence intervals.

### Theoretical Considerations for Temperature Scaling of TK–TD Parameters

An effect of temperature on the TK–TD model parameter has been assumed previously,^10^ although not evaluated quantitatively (but see Heugens et al.^9^). As a starting point, Jager and Ashauer^10^ discuss the general assumption applied in the dynamic energy budget theory, where all physiological rates scale with temperature in the same way,^18^ while pointing out the open question for transferring this approach to the GUTS framework. Here, we provide a brief reflection of the theoretical consideration for temperature scaling of TK–TD parameters, while a detailed rationale for each parameter corrected (or not corrected) for temperature is provided in Supporting Information 01.

With increasing temperature, chemical reactions accelerate, with their rates often following the Arrhenius equation.^15^ Within this concept, temperature influences the time axis by increasing or decreasing the reaction rate, i.e., speeding up or slowing down the process, respectively. Thus, starting with the most straightforward approach, we can assume that all rates (i.e., parameters that include the dimension of time) scale with temperature. This includes all TK rates, the damage repair rate (*k*_r_), the background hazard rate (*h*_b_), and the killing rate (*b_i_*) of the SD mechanism. Due to the simplifications (or assumptions) made to construct the model, particularly for the central damage concept, it is not trivial to translate the potential mechanisms behind this damage to the real-life scenario. This depends on the compound and the organism.

The mechanism of toxic action for neonicotinoids and other nicotinic acetylcholine receptor (nAChR) agonists, like FPF, has been described in considerable detail.^20−24^ Despite this detailed description (see Supporting Information 01) of the adverse outcome pathway (AOP) of IMI, which we also deem to be applicable for FPF, quantifying these processes explicitly is beyond the scope of this research. Nonetheless, the generic damage concept of GUTS indirectly integrates those processes through fitting the measured survival. However, we would like to emphasize that the considerations for the temperature scaling of the TK–TD parameter as further elaborated in Supporting Information 01, though based on the mechanistic understanding of the AOP for neonicotinoids, are theoretical and not quantitatively confirmed. As such, these considerations should be treated with care when applied to other compounds, organisms, and exposure scenarios.

## Results

### Toxicokinetic Modeling

#### Disregarding Temperature in TK Modeling (TK)

**Figure 2:**
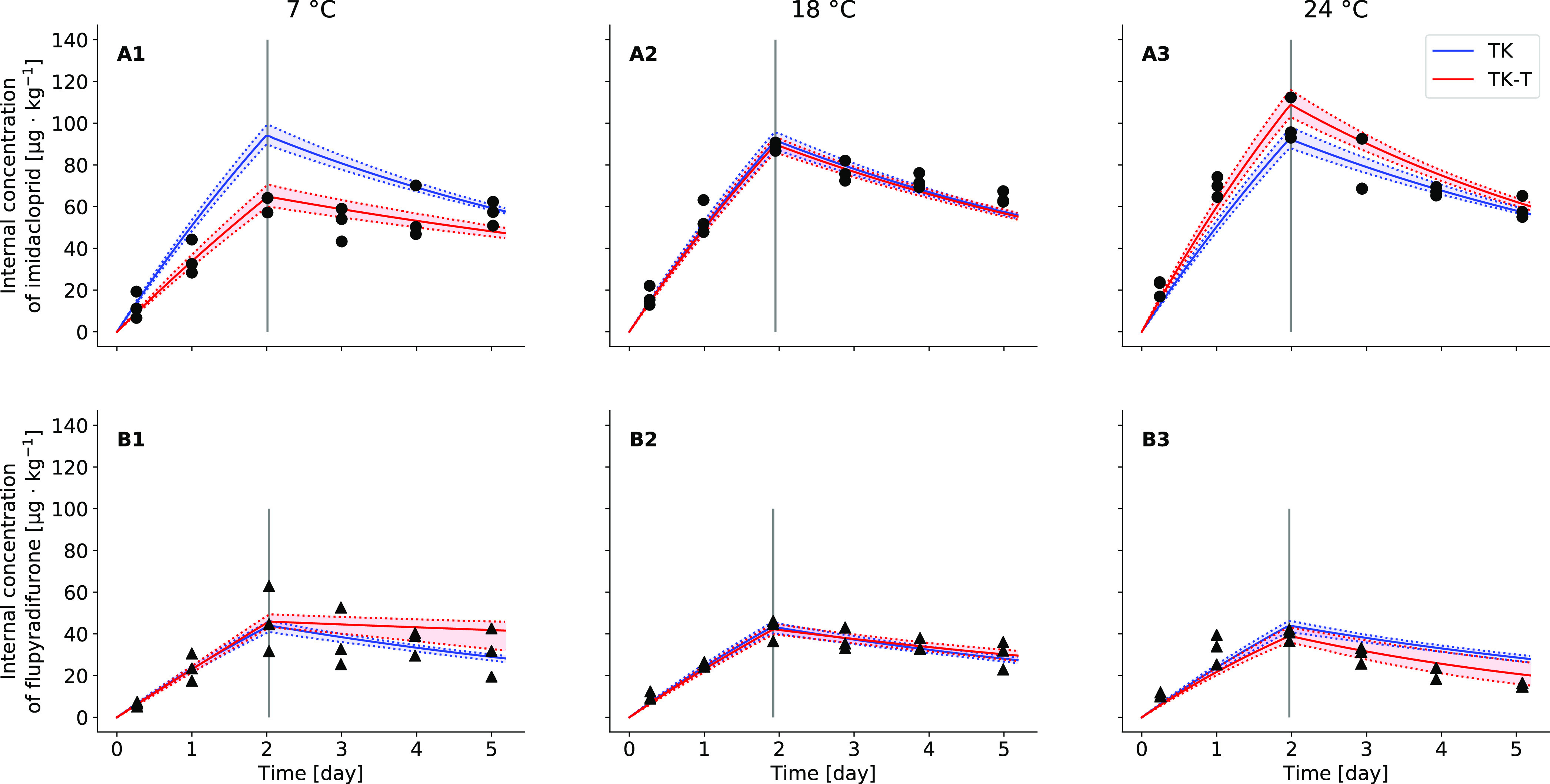
Internal concentration of insecticides in *Gammarus pulex* at different temperatures. Black symbols are measured internal concentrations at 7, 18, and 24 °C, respectively. Blue solid lines are the TK model fits (without temperature correction), and red solid lines are TK-T model fits (with temperature correction) with lower and upper confidence intervals (dotted lines). The vertical gray line marks the transition time point from uptake to elimination phase for each temperature and insecticide. Upper panels A1–A3: imidacloprid; lower panels B1–B3: flupyradifurone.

When using the data sets to calibrate the TK model without considering the experimental temperatures (i.e., treating the data sets as replicates), the model failed to capture the measured internal concentrations for the low and high temperatures (Figure 2, blue lines). The TK approach overestimated the internal concentrations of IMI at 7 °C and slightly underestimated them at 24 °C. Reasonably, the model fitted the medium temperature (18 °C) well for IMI, as the measured internal concentrations were in between those from the low and the high temperatures. Similarly, for the FPF data sets, we observed a good fit at 18 °C. Due to the high variation of measured concentrations, the model was within the measured values at 7 °C, whereas it overestimated the internal concentration during the elimination phase at 24 °C.

#### Temperature Explicit TK Modeling (TK-T)

For the TK-T model, temperature was explicitly considered in the calibration through an additional parameter, the Arrhenius temperature *T*_A_. Comparing the TK-T with the TK model where temperature is not considered, the TK-T results were a better fit, with an AIC difference of 37.9 for IMI and 5.4 for FPF (Supporting Information 02, Table S3).

For the IMI data, the profile likelihoods for the individual parameters were well-defined (Supporting Information 01, Figure S3, plots on the diagonal). Thus, all model parameters and their CIs were identified (Supporting Information 01, Table S2). However, the likelihood-based joint-confidence regions showed a high correlation of model parameters, specifically *k*_e_ and *k*_em_, visible in their narrow shaped bounds (Supporting Information 01, Figure S3).

During the TK-T model calibration for the FPF data, it was noticed that the parameter boundaries for *T*_A_ were not significantly different from zero when correcting both rates (*k*_u_ and *k*_e_) with the Arrhenius equation (Supporting Information 01, Figure S4). As *k*_u_ for FPF did not differ substantially across temperatures, the TK-T approach could not fit the parameter *T*_A_. Thus, when finally calibrating the TK-T model for the FPF data set, we did not correct *k*_u_ for temperature, but only *k*_e_, which resulted in well-defined confidence regions for *T*_A_ (Supporting Information 01, Figure S5). Furthermore, the model with only *k*_e_ corrected for temperature resulted in a significantly better fit, i.e., it had an AIC value of 417 compared to 425 for the model with both rates corrected for temperature. An alternative method to address the different temperature sensitivity of the rates would be to introduce separate *T*_A_ values for uptake and elimination (i.e., *T*_A_-*k*_u_ and *T*_A_-*k*_e_). Though this approach would likely result in a good fit capturing the temperature changes of both rates individually, it would also increase the risk of model overfitting or nonidentifiability. We decided to favor the model version with fewer parameters.

### Effect Modeling

#### Explicit Consideration of Temperature in Effect Models

**Figure 3:**
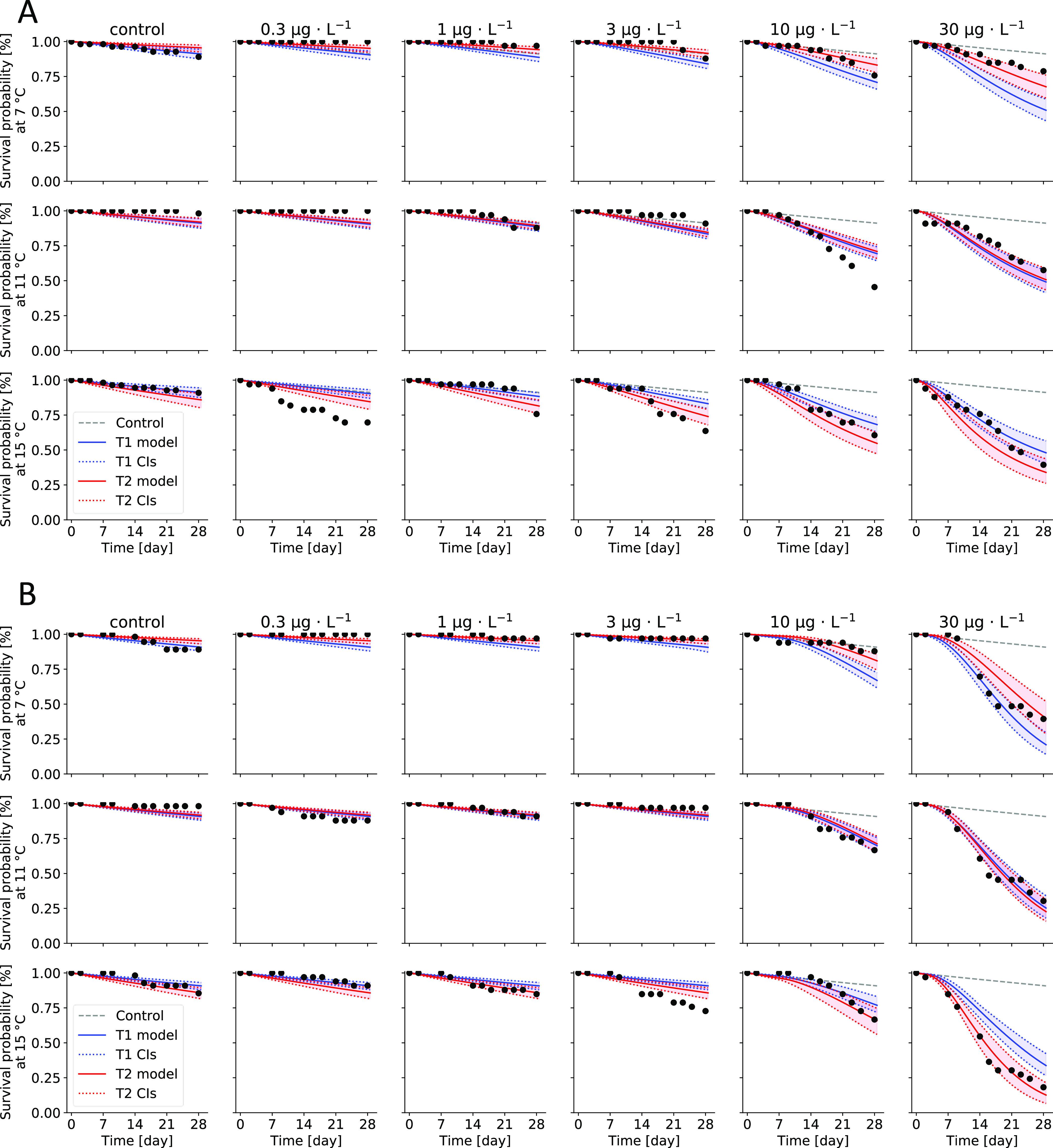
Concentration–response curves for the survival of *Gammarus pulex* exposed to imidacloprid (A) and flupyradifurone (B) at different temperatures. Black dots represent the measured survival (replicates pooled) at the different exposure levels in μg·L^–1^ (column headings) and the different temperatures in degree Celsius (*y*-axis label). The blue line shows the GUTS-FULL-T1 model (i.e., only toxicokinetic parameters corrected for temperature) prediction for survival, and the dotted blue lines show the boundaries of its 95% confidence interval (blue area). The red line shows the GUTS-FULL-T2 model (i.e., also toxicodynamic parameters corrected for temperature) prediction for survival, and the dotted red lines show the boundaries of its 95% confidence interval (red area). The gray dotted line shows the control mortality as modeled under GUTS-FULL-T1 for each temperature. For IMI, the IT models are shown and for FPF, the SD models.

On visual examination, the model calibrations for GUTS-FULL-T1 (i.e., only TK parameters corrected for temperature) and GUTS-FULL-T2 (i.e., also TD parameters corrected for temperature) resulted in reasonable fits to the survival data of *G. pulex* exposed to IMI or FPF (Figure 3). For IMI, the survival probability estimated by both models at 11 °C overestimated the measured survival in the 10 μg·L^–1^ treatment, which is related to the experimental data showing no difference between 10 and 30 μg·L^–1^ treatments. Furthermore, the measured survival at 15 °C in the 0.3 and 3 μg·L^–1^ treatments showed a similar pattern as the 10 μg·L^–1^ treatment, which was not well captured by both model fits. Again, the observed survival over time shows no systematic influence of the concentration except for the highest treatment level, so the TK–TD modeling could not fit the observed variations in the lower treatments, which might be predominantly related to variation in background mortality. Slightly better model performance was achieved for the survival data of *G. pulex* exposed to FPF. Here, the survival in the 10 μg·L^–1^ treatment at 7 °C was underestimated and overestimated in the 3 μg·L^–1^ treatment at 15 °C by both models. When focussing on the two highest concentrations tested, the different model applications overlap at the intermediate temperature (11 °C) for both chemicals (Figure 3 center panels). While the GUTS-FULL-T1 (blue curves) approach underestimated the survival in lower temperatures and overestimated it in higher temperatures, GUTS-FULL-T2 (red curves) fitted the survival data obtained at 7 and 15 °C better for both chemicals (Figure 3).

The AIC differences between the model approaches also support GUTS-FULL-T2 as the better-performing model. With an AIC difference of 21 for the IMI models and 27 for the FPF models, GUTS-FULL-T2-SD overall performed significantly better than GUTS-FULL-T1-SD (Table 1). The same conclusion was obtained for the IT models with an AIC difference of 22 for IMI and 23 for FPF (Supporting Information 02, Table S3). Overall, the AIC value evaluation revealed GUTS-FULL-T2-IT to be the best performing model for IMI and GUTS-FULL-T2-SD for FPF (Table 1).

**Table 1:** Toxicodynamic Model Parameters for *Gammarus pulex*^a^

		imidacloprid				flupyradifurone			
**GUTS-FULL-T1**		stochastic death		individual tollerance		stochastic death		individual tollerance	
parameter	unit	value	95% CI	value	95% CI	value	95% CI	value	95% CI
*k*_r_	d^–1^	0.069	(0.002–10^b^)	0.001^b^	(0.001^b^ – 0.017)	1.714	(0.327–10^b^)	0.082	(0.006–0.202)
*m*_i_	μg·kg^–1^	0.001^b^	(0.001^b^–9)	12	(8–182)	69.0	(39–101)	284.3	(38–412)
*h*_b_	d^–1^	0.004	(0.003–0.005)	0.003	(0.002–0.005)	3.41^–03^	(2.51^–03^–0.004)	0.003	(0.002–0.004)
*b*_i_	kg·μg^–1^·d^–1^	1.28^–04^	(5.51^–05^–0.007)			1.40^–04^	(1.02^–04^–1.87^–04^)		
Fs	[−]			42	(18–107)			8	(5–15)
AIC	[−]	1218.40		1219.26		1129.12		1124.72	
**GUTS-FULL-T2**									
*k*_r_(T)	d^–1^	0.252	(0.001^b^–10^b^)	0.001^b^	(0.001^b^–0.052)	8.372	(1.079–10^b^)	0.145	(3.61^–05^–0.590)
*m*_i_	μg·kg^–1^	1.00^–6^^b^	(1.00^–6^^b^–9.807)	3.4	(2–148)	81.9	(50–108)	184.2	(0.1^b^–333)
*h*_b_(T)	d^–1^	0.009	(0.006–0.016)	0.011	(0.006–0.019)	0.011	(0.007–0.017)	0.009	(0.005–0.016)
*b*_i_(T)	kg·μg^–1^·d^–1^	2.41^–04^	(1.31^–04^–0.031)			5.56^–04^	(3.16^–04^–9.46^–04^)		
Fs	[−]			41	(19–101)			8	(5–15)
*T*_A_-td	K	8510	(4690–13170)	12150	(7189–17610)	11730	(7342–15910)	11310	(6919–16140)
AIC	[−]	1196.91		1196.82^c^		1101.41^c^		1101.82	

a The parameters were estimated for a reference temperature (*T*_ref_) set to 20 °C. The respective toxicokinetic model parameters can be found in Supporting Information 01, Table S2. Parameter symbols are explained in Supporting Information 01, Table S1. CI = confidence interval, AIC = Akaike’s information criterion.

b Boundary of the parameter space explorer.

c Lowest AIC.

Considering the likelihood-based joint-confidence regions for the model parameter (Supporting Information 01, Figures S8–S15), potential identifiability problems are apparent for both approaches, i.e., GUTS-FULL-T1 and GUTS-FULL-T2. For IMI, the method revealed identifiability problems for the damage repair rate (*k*_r_) parameter in all models and the median threshold for survival (*m_i_*) in SD models (marked with an asterisk in Table 1).

The exposure of *G. pulex* to FPF caused an effect on the survival, mainly in the two highest concentrations tested (Figure 3B). Here, the model parameter’s profile likelihoods presented overall well-defined parabolic shapes (Supporting Information 01, Figures S10, S11, S14, and S15) and identified internal threshold values as significantly different from zero. For both the GUTS-FULL-T1 and the GUTS-FULL-T2 approach, *k*_r_ ran into the set boundaries of the parameter space explorer for SD models (Table 1 and Supporting Information 02, Table S2) and was here as well correlated strongly to the internal threshold values m_i_, which ran into the lower boundary in the GUTS-FULL-T2-IT model.

#### Temperature Influences the Toxicity of Insecticides

**Figure 4:**
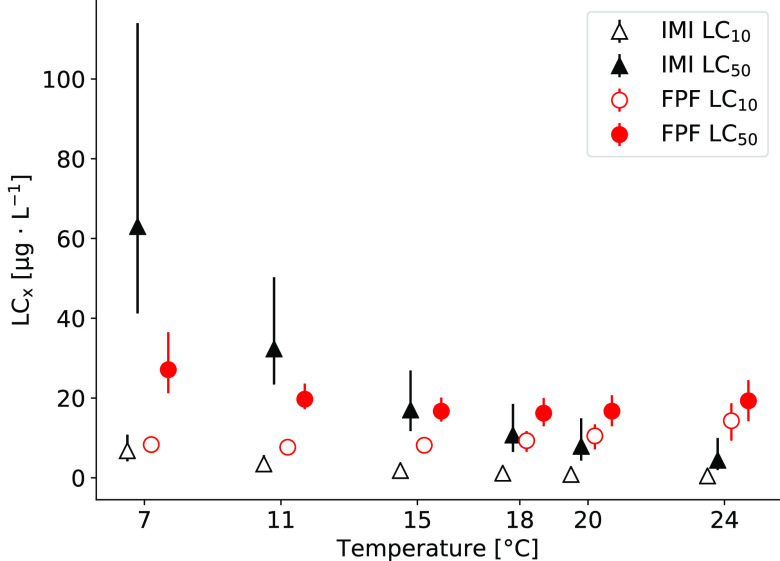
Simulated lethal concentrations (LCx) for insecticides at different temperatures after 28 days. Solid shapes for LC_50_ of imidacloprid (IMI, black triangles) and flupyradifurone (FPF, red circle) and empty shapes for LC_10_, respectively. Error bars represent the 95% confidence intervals based on the parameter sets between the dotted horizontal lines of the profile likelihoods of Figure S13 for IMI and Figure S11 for FPF.

With the calibrated parameters of the best-fitting models (Table 1), we predicted a 28-day LC_50_ of 7.89 (95%-CI: 4.32–14.9) μg·L^–1^ for IMI and 16.7 (95%-CI: 12.9–20.7) μg·L^–1^ for FPF at the reference temperature (20 °C). The LC_50_ predictions at 7, 11, 15, 18, 20, and 24 °C showed a different pattern for both insecticides (Figure 4). While LC_50_ and LC_10_ values for IMI decreased with increasing temperature, and remained the same ratio, the LC_50_ values for FPF slightly decreased from 7 to 18 °C and increased again from 20 to 24 °C (Supporting Information 02, Table S4). The LC_10_ for FPF decreased from 7 to 11 °C and then increased with rising temperatures from 15 to 24 °C.

## Discussion

### Toxicokinetic Modeling

#### Explicitly Considering Temperature Improved the TK Modeling of IMI and FPF in *G. pulex*

Our results showed an evident temperature influence on the TK of both insecticides. This temperature influence was well captured by the TK-T model calibration (Figure 2), supporting the use of the Arrhenius equation to correct for temperature effects on toxicokinetic processes. Furthermore, the previously calibrated TK parameter in Huang and Mangold-Döring et al.^14^ can be used for comparison. It is important to emphasize that the TK model calibrations on the same data sets in this previous study^14^ were done separately for each experimental temperature, i.e., implicitly accounting for temperature. In contrast, the TK-T calibration in the present study accounted for temperature explicitly via the Arrhenius equation using all three temperature data sets simultaneously. Comparing the two different model fits, there is almost no difference (Supporting Information 01, Figure S1). Thus, by explicitly accounting for temperature in the TK-T model, it is possible to describe the toxicokinetic processes of IMI and FPF in *G. pulex* by one parameter set per chemical (Supporting Information 01, Table S2), in contrast to three separate factors parameter sets in the conventional approach previously used.^14^

However, the observed temperature influence in the previous study was different between the uptake and the elimination rates (i.e., for IMI, a 2.1-fold difference of *k*_u_ and a 4.9-fold difference of *k*_e_) and between the two insecticides (with only a 1.3-fold difference for *k*_u_ and a 3.1-fold difference of *k*_e_ for FPF). Thus, with the data currently available for the uptake kinetics of FPF, we found no significant scaling with temperature (also see the reasoning in the Results section) and therefore treated this rate to be constant in the TK-T approach. Even though the fit to the data supports removing the temperature correction for *k*_u_ in the FPF model, this is no proof that the uptake processes of FPF are not affected by temperature. Assuming diffusion to be the main uptake route, a temperature influence on the uptake is to be expected, although not represented in our data. However, for the elimination kinetics, the 3.1-fold difference was well captured through the correction with the Arrhenius equation (Figure 2).

Furthermore, the scaling of the TK processes with temperature appeared to be different between the two chemicals. While the confidence interval of the *T*_A_-tk overlapped, the best-fitting value for IMI, with 3044 K, was only a third of the value for FPF with 9243 K (Supporting Information 01, Table S2). The larger confidence interval (95% CI: 1943–15870 K) for FPF compared to IMI (95% CI: 2316–3724) is likely related to the limited information in the data for FPF, i.e., *T*_A_-tk was fitted to the elimination kinetics only.

Increased uptake of insecticides with increasing temperature was reported in previous studies for aquatic invertebrates.^8,25−28^ The predominantly proposed mechanism for this result is the increase in the organism’s metabolic activity with rising temperatures. A higher metabolic activity demands an increase in oxygen supply, which the organism may achieve by increasing its ventilation rate, simultaneously increasing the uptake of contaminants present in the water.^25,26^

Elimination processes mediated by enzyme activity (e.g., biotransformation) will likely also show an increase with increasing temperature, at least up to the enzyme’s temperature optima. As reported in Huang and Mangold-Döring et al.,^14^ the biotransformation of IMI to IMI-ole increased 2.2-fold from 18 to 24 °C, also visible in the TK-T model fits of this study (Supporting Information 01, Figure S2). Unfortunately, the temperature influence on elimination rates appeared to be understudied for aquatic organisms. However, the few available studies show an increase with increasing temperature, for cadmium,^29^ pyrethroid insecticides,^8^ and persistent organic pollutants,^30^ in accordance with the present study of IMI and FPF.

### Effect Modeling

#### Correcting TK and TD Parameters for Temperature Results in a Better Fit of GUTS

A parameter identifiability problem^31^ arises when the observed data sets do not hold enough information (e.g., too little difference in effects between concentration levels tested) to properly determine one or more model parameter and their confidence intervals. A lack of information can result in a model parameter not being differentiated from zero; therefore, the lower confidence limit cannot be adequately determined. Thus, the observed identifiability problems for the model parameters in estimating their likelihood-based joint-confidence regions are probably related to the low effect sizes observed during the 28-day exposure, i.e., significant effects only in 10 and 30 μg·L^–1^ treatments (Figure 3). Another reason for identifiability problems to occur is when there is “slow kinetics”, as discussed in the GUTS e-book by Jager and Ashauer.^10^ In this case, the compound’s dynamics are slow compared to the exposure duration, resulting in the parameter boundaries of k_e_ and m_i_ going toward zero and b_i_ going toward infinity (for the SD model), and those parameters correlating with each other.

From comparing the distinctive calibration approaches of the GUTS-FULL models, we conclude that GUTS-FULL-T2 (i.e., TK and TD parameters corrected for temperature) explains the variation in the data under different temperatures best for both insecticides. Therefore, only accounting for the temperature influence on TK parameters (i.e., GUTS-FULL-T1) is, although possible, less appropriate to model insecticides’ toxicity at different temperatures. Thus, our study suggests that TD processes are also affected by temperature. It should be noted that the difference between both applications mainly arises from the two highest concentration levels. In support of this result, additional toxicity experiments with more distinctive effect levels are needed. Nevertheless, our results support the explicit consideration of temperature in GUTS-FULL applications with the help of the Arrhenius expression, as presented in our GUTS-FULL-T2 approach.

#### Differences in Temperature Scaling of TK and TD Model Parameters

The GUTS-FULL-T2 approach fits only one Arrhenius temperature (*T*_A_-td) to correct all TD parameters simultaneously. This approach was conducted in line with the argumentation for a single (species-specific) *T*_A_ that scales all metabolic rates, as maintaining homeostasis would otherwise be troublesome for the organism.^18,32^ We applied the same argumentation to the TK parameters, which were scaled with one *T*_A_-tk. Hence, in this approach, it cannot be determined if single parameters might also change with temperature differently. We refrained from evaluating this question in the current study, as the observed effects in the available data sets were predominantly in the two highest concentrations, limiting the information needed to identify single parameters’ temperature sensitivity successfully. However, Heugens et al.^9^ observed that parameters were not affected by temperature in the same way. Thus, it remains to be determined which TD parameter is most sensitive to temperature and if a single *T*_A_-td applied to all parameters is sufficient. For TK rates of organic contaminants, Raths et al.^33^ showed that the uptake and elimination rates scale with a similar *T*_A_ when evaluated separately with the Arrhenius equation, supporting the approach used in this study.

The Arrhenius temperatures for TK and TD were considerably different for IMI but more similar for FPF, due to the large confidence interval (Table 1 and Supporting Information 01, Table S2). With 3044 K (2316–3724 K) for IMI and 9243 K (1943–15 870 K) for FPF, *T*_A_-tk values were smaller than *T*_A_-td values for IMI 12150 K (7189–17 610 K) and FPF 11730 K (7342–15 910 K). This result indicates that the temperature scaling described by the Arrhenius equation is considerably different for processes of TK and TD, especially for IMI. Furthermore, it would be interesting to investigate if the temperature scaling of TK processes for insecticides is similar across aquatic organisms. Using the uptake rates of IMI in *Isonychia bicolor* reported by Camp and Buchwalter,^26^ we could obtain a *T*_A_-tk of 4700 K. Though this value is close to the *T*_A_-tk value in the present study, it remains unclear if this parameter can be applied to correcting the TK rates of IMI across species or chemicals. Looking for the same comparison on the TD side, there are no literature values available. As the TD processes in the GUTS approach are generally related to an abstract internal damage state, they cannot be associated with a specific effect mechanism. However, it is possible to determine a *T*_A_ based on physiological processes, e.g., growing and aging, with the Add-my-Pet (AmP) tool (https://add-my-pet.github.io/AmPtool/docs/index.html) following the dynamic energy budget theory.^18^ With the entries for *G. pulex* in the AmP collection, a *T*_A_ of 10556 K is obtained (Zimmer et al.,^34^ parameter estimated based on code version 20210703). Interestingly, the *T*_A_-td values in the present study are within the range of the *T*_A_ derived from the AmP collection. This could indicate that the TD processes reflected in GUTS are affected by temperature in the same way as the physiological processes, i.e., growth, reproduction, and aging.

#### Insecticide-Specific Increase or Decrease of Toxicity with Temperature

The predicted LC*_x_* values based on the best-fitting GUTS-FULL-T2 models differed at the various temperatures for both insecticides (Figure 4). For FPF, LC_50_ values first decreased from 7 to 18 °C as observed for IMI but then increased again in higher temperatures. As for IMI, the uptake and the elimination rates in TK-T increase with rising temperatures, and the LC_50_ and LC_10_ values show a constant decrease. However, the uptake rate for FPF in the TK-T approach was kept constant for discussed reasons, which explains the increase in LC_x_ values for FPF. With a constant uptake rate (*k*_u_) but the elimination rate (*k*_e_) scaling with temperature, there is a point where the elimination of FPF is faster than its uptake, resulting in a lower toxicity (i.e., a higher LC*_x_*).

For comparison, we can consult the results of the previous analysis of the measured survival data.^14^ The LC_10_ and LC_50_ values obtained from the concentration–response curve analysis based on a log-logistic regression decreased with increasing temperature for both compounds, suggesting increased toxicity of both compounds with increasing temperature in the range of 7–11 °C, in line with the analysis in this study. The differences in the obtained values are associated with the different analysis approaches. The regression analysis fits the model curve only to the survival data of each temperature data set separately, while the GUTS-FULL approach presented in this study considers survival and internal concentration measures based on the mechanistic assumptions underlying the GUTS framework using all data. Nevertheless, in both results, the influence of temperature on the toxicity differs for the two compounds (i.e., effects do not scale with temperature the same).

These findings highlight the importance of further investigation of the chemical-dependent influence of temperature on the toxicity mechanism of insecticides, even when they have the same molecular target, i.e., for IMI and FPF, the binding to the organism’s nAChRs.^21,22^ A good starting point to investigate the temperature-dependent toxicity of insecticides with the same molecular target could be their binding affinities. For example, for pyrethroid insecticides (i.e., sodium channel modulators),^35^ it has been observed that the binding to their target site is higher in lower temperatures,^36^ increasing their toxicity.^8^ However, as the evaluation of temperature influence on binding affinities of neonicotinoids to nAChR has not been investigated, further studies are required.

#### Advantages, Usefulness, and Limits of the Temperature Explicit TK and GUTS Models

By explicitly considering the influence of temperature on TK–TD processes, the internal concentrations of an insecticide and its effect on organism survival can be predicted for various temperature settings. However, it is important to note that the Arrhenius expression only applies within the species’ thermal window. For temperatures beyond the lower and upper critical temperatures, the exponential relationship inherent to the Arrhenius equation may lose its applicability, i.e., organisms may reduce metabolic activity; thus, physiological rates are likely to decrease. Therefore, when predictions outside the thermal tolerance range of an organism are necessary, other temperature models^37,38^ should be tested, as outlined in the supporting information of Goussen et al.^39^ Nevertheless, the temperature explicit mechanistic effect models as presented in this study hold the potential to investigate further the underlying mechanisms of temperature effects on the toxicity of chemicals in other environmental scenarios, i.e., daily temperature fluctuations.^40^

The temperature explicit models evaluated in this study provide a parameter set at a reference temperature (here 20 °C), enabling a temperature neutral comparison for various insecticides’ uptake and elimination properties and their toxicity. Furthermore, we showed that data obtained for the same species at different temperatures could be combined to parameterize the temperature explicit models, which will improve cross-study evaluations. The results of our experimental and modeling studies on the influence of temperature on the adverse effects of insecticides in aquatic organisms show the relevance of temperature for the observed effects. This study demonstrates that applying mechanistic effect models to experimental data gains insights beyond the standard toxicity information. While GUTS modeling provides the advantage of understanding toxicity in time, this study expands the understanding to the additional dimension of temperature influences on toxicity, i.e., which processes governing toxicity are affected by temperature. In our analyses, these insights are restricted by the model’s mechanistic details and assumptions. The damage concept of GUTS remains a simple black box, where critical molecular processes are not explicitly considered without further knowledge of the contaminant’s mode of toxic action. By extending the GUTS framework with these chemical and organism-specific processes, a more detailed analysis of those underlying processes is possible. Thus, attempts to connect generalized mechanistic concepts as the adverse outcome pathways with effect models such as GUTS^41^ can be used to open the box and fully understand the mechanisms behind contaminants’ effects on organisms and how temperature modulates it.

## Supporting Information Available

Supporting Information 01: Text document holding the rationale for temperature correction of the TK–TD parameter, model equations, and additional figures from the analysis (PDF)
Supporting Information 02: Spreadsheet with all TK–TD model parameters, AIC comparison, and LC*_x_* predictions (XLSX)
