# Supplementary material for: Explicit Consideration of Temperature Improves Predictions of Toxicokinetic–Toxicodynamic Models for Flupyradifurone and Imidacloprid in Gammarus pulex
Source: Environ Sci Technol. 2022 Oct 25;56(22):15920–9. doi: 10.1021/acs.est.2c04085 (PMC9671055; doi:10.1021/acs.est.2c04085)
Supplement: Supplementary file 1 — es2c04085_si_001.pdf [file es2c04085_si_001.pdf]

Supplemental Information 01 for the Article:

Explicit consideration of temperature improves predictions of toxicokinetic-toxicodynamic models for flupyradifurone and imidacloprid in *Gammarus pulex*

Authors:

Annika Mangold-Döring[1]*, Anna Huang[1], Egbert H. van Nes[1], Andreas Focks[2], Paul J. van den Brink[1,3]

*annika.mangold-doering@wur.nl

Affiliations:

[1]Department of Aquatic Ecology and Water Quality Management, Wageningen University and Research, P.O. box 47, 6700 AA Wageningen, the Netherlands

[2]System Science Group /Institute of Mathematics, Osnabrück University, Barbarastr. 12, D-49076 Osnabrück, Germany

[3]Wageningen Environmental Research, P.O. Box 47, 6700 AA Wageningen, the Netherlands

Tables: 2

Figures: 15

Pages: 21

Theoretical considerations for temperature scaling of TKTD parameters

As chemical reactions accelerate with increasing temperature, we argue that temperature influences the time axis by increasing or decreasing the reaction rate, i.e., speeding up or slowing down the process, respectively. With this, we can assume that all rates (i.e., parameters that include the dimension of time) scale with temperature. This includes all TK rates, the damage repair rate (k_r), the background hazard rate (h_b), and the killing rate (b_i) of the SD mechanism. While this assumption can easily be applied to the model equations, the translation from the mathematics to the biological processes in reality, is not so straightforward. This is due to the simplifications (or assumptions) made to construct the model, leaving some uncertainties regarding translation to real-life processes. This challenge is particularly difficult for the central damage concept integrating a variety of different processes leading to an increased hazard for the organism. Translating the potential mechanisms behind this damage to the real-life scenario depends on the compound and the organism. In the following, we elaborate on this in generall, as well as on the example of *Gammarus pulex* exposed to nAChR (nicotinic acetylcholine receptor) agonists.

Toxicokinetics (TK)

The toxicokinetic models used in this study can be defined as first-order one-compartment models. They represent an organism as one well-mixed compartment in which the chemical is taken up, possibly metabolized (or biotransformed), and eliminated. Like all models, this model simplifies reality and does not distinguish between the individual processes behind the overall uptake kinetic as defined in the model equations (eq. S2a-c). In other words, although the TK model parameters are fitted to the real measures of internal concentration in the organism, we do not know how exactly this uptake happened. However, we can make assumptions regarding those underlying processes and how they could be influenced by temperature. To follow the simplest approach, we assumed the Arrhenius temperature (T_A-tk) to be the same for all TK parameters. Probably this is not always correct, but the quality of the data limits the number of parameters that we can fit independently.

Uptake rate (k_u). Freshwater organisms like *Gammarus pulex*, which spend their whole life cycle in the water, are exposed to compounds solved in the water phase (aqueous exposure), absorbed into the sediment, or adsorbed to detritus which serves as food. Thus, potential uptake routes are various. As the TK experiments in this study took place in a sediment-free setup, without feeding during the exposure period, we can safely assume that the uptake of the compounds was limited to the aqueous phase (note: however, there was feeding with leaves in the chronic toxicity experiment). Thus, the main route of uptake for water-soluble chemicals like imidacloprid (IMI) and flupyradifurone (FPF) is passive diffusion across the gills[1], which are in direct contact with the aqueous phase. Diffusion itself is influenced by temperature due to the temperature-dependent movement of molecules (i.e., increasing movement with increasing temperatures).[2] However, this physicochemical process is probably negligible compared to the effect of increased ventilation due to higher oxygen demand in higher temperatures.[3] Oxygen uptake is driven by passive diffusion through the concentration gradient at the gill-water interface.[4] Dissolved oxygen concentration in water decreases with higher temperatures; thus, with less oxygen in the water, more water must pass through the gills to supply enough oxygen. *G. pulex* can achieve this by increasing the beating of its pleopods.[4] With more water passing through the gills to supply oxygen, the compounds solved in the water also pass through the gills, resulting in increased uptake rates in higher temperatures.[5] Though we did not quantify the oxygen consumption of the organisms in the present study, the dissolved oxygen concentrations in the experimental units were measured, decreasing with increasing experimental

temperatures from 9.9 to 8.9 mg $O_2 \cdot L^{-1}$ in week 4 (see water parameters in Mendeley data[6] of Huang and Mangold-Döring et al. 2022).[7]

Elimination rate of the parent compound (k_e) and the metabolite (k_{em}). Per definition, the elimination rate describes the speed of the compound exiting the defined compartment (i.e., the organism). The biological processes defining the elimination can certainly include the same routes as the uptake (i.e., diffusion across the gills) and thus would be influenced by temperature in the same way. Additionally, elimination via urine and feces is possible. When evaluating these routes, the parent compound likely undergoes biotransformation (next paragraph). If biotransformed, the parent compound does not eliminate from the compartment by exiting it but gets degraded into biotransformation products (often called metabolites). Overall, the elimination rates k_e and k_{em} are expected to increase with increasing temperature.

Formation rate of the metabolite (k_m). Enzymes have an optimal temperature in which their catalytic performance is maximized. Thus, biotransformation processes mediated by enzymes will likely increase with increasing temperature, given that the temperature increases towards the temperature optimum and not beyond. Increased biotransformation processes will generate more metabolites (i.e., biotransformation product). We can thus expect an increase in the formation rate of the metabolites with increasing temperature. In the specific case of neonicotinoids, their biotransformation in insects is mediated by microsomal cytochrome P450 monooxygenases.[8,9]

Toxicodynamics (TD)

Within the toxicodynamic part of the GUTS-FULL model, the observed survival over time is used to fit the model parameters. Within the TD part, the central concept is the damage compartment, which remains a black box, especially without further definition of the particular organism and compound combination. Briefly, the damage caused by the compound can be accumulated over time inside the organism, and this damage can potentially be repaired over time (i.e., recovery). The reasoning why this black box is useful and a listing of the underlying assumptions can be found in the handbook for GUTS by Jager and Ashauer.[10] Building on this, we here discuss the potential underlying TD processes for the specific cases of IMI and FPF and how they may or may not be influenced by temperature. In the TD part we also assumed one Arrhenius temperature (T_A-td) for all TD parameters.

IMI and FPF are classified as competitive modulators for the nAChR.[11] The damage caused by such compounds arises from their binding to the nAChR.[12,13] This binding initiates a chain of events, starting with the conformation change of the receptor that promotes the influx and efflux of calcium ions (Ca^{2+}), extracellular sodium (Na^+), and intracellular potassium ions (K^+), creating an action potential for synaptic signalling.[14–16] Under normal conditions, this conformation change of the receptor is induced by the neurotransmitter acetylcholine (ACh), which also initiates the release of its counterpart, the acetylcholinesterase (AChE). AChE then hydrolyzes ACh, reversing the conformation change and thus inhibiting the ion flow, which terminates the impulse transmission at cholinergic synapses.[17] As this hydrolyzation does not occur for the nAChR agonists[18] IMI and FPF, they cause a biphasic response.[12] As described by Maloney 2020[13], the first phase is determined by the excitement of the: "cholinergic neurons/myocyte, increasing the frequency of spontaneous discharge, resulting in uncontrollable muscle tremors, cell energy exhaustion, and cell death[12,16]". In the second phase, the neurons get desensitized to the natural agonist ACh, which impedes the propagation of naturally induced impulses. This loss of normal neuromuscular function causes paralysis and eventually death.[12,13,18,19]

Damage-repair rate (k_r). This parameter describes the processes of damage repair and thus can be interpreted as the "elimination rate of damage." If we assume that enzymes mediate the repair mechanism, we can argue for its temperature dependence similar to the parameter k_m. For IMI and FPF, a possible active (i.e., enzyme-mediated) repair mechanism could be the metabolization of the parent compound (i.e., eliminating the nAChR blockades described above). Thus, the temperature dependence of the damage repair rate can be derived from the temperature dependence of the enzymes for biotransformation (i.e., increasing enzyme activity with increasing temperature).

Additionally, active repair mechanisms (i.e., building new nAChR, formation of new cells) could benefit from increased temperature (i.e., access energy in the form of heat, decreasing activation energies for bio-chemical transformations). Besides, if the damage repair rate is high, we can expect that the damage level is closely related to the compound's internal concentration. Thus, the damage repair is so quick that the damage dynamics follow the TK. The damage level will lag behind the compound dynamics with a low damage repair rate. Generally, we thus assume the damage repair to increase with increasing temperature.

Median (m_i) and spread factor (F_s) of the threshold distribution. In GUTS, the chemical needs to exceed a certain threshold (m_i) of the damage level to contribute to the actual organisms' hazard on survival. Within an (experimental) population, the thresholds for the individuals are defined by a single value in the SD model (i.e., m_i) or follow a log-logistic distribution in the IT model, defined by the median (m_i) and the spread factor (F_s). Thus, these parameters are not very likely to scale with temperature. Assuming that m_i would scale with temperature, the median threshold would get higher. Translating this back to reality would mean the organisms would become less sensitive. For an infinite timescale, one could argue that this could be achieved due to acclimatization processes and evolution. But to our knowledge, there is no explanation for a reduced sensitivity with increased temperature over short periods (i.e., less than one month, as in our experiments).

Similarly, we argue for the threshold distribution's spread factor used in the individual tolerance (IT) death mechanism of GUTS. If F_s increased with temperature, the distribution of the thresholds would widen, meaning the organisms would become more different in their sensitivity. Again, we could not think of any reason for such behavior and thus refrained from adjusting mi and F_s for temperature, assuming those parameters to be temperature independent.

Background hazard rate (h_b). Taking the chemical-induced stress aside, each individual has a certain probability of dying. In the context of laboratory experiments, this probability can include the handling of the individuals or other random processes which are usually not nearer defined, other than explicitly excluding the influence of chemical stress. This parameter is thus fitted to the mortality occurring in the control treatments. In the chronic toxicity experiments used for the model calibrations of this study, the temperature was present in all treatments, including the controls. If temperature itself would affect the survival of the organism, we would thus see it in the control treatments. Even if the narrow temperature range used in the chronic exposures is not expected to influence the organisms' survival, the temperature may increase the background hazard rate due to inducing thermal stress. Thus, we can expect the parameter h_b to increase with increasing temperature.

The killing rate (b_i). As given by its name, this parameter determines the speed of death within the GUTS model. Used only for the stochastic death (SD) mechanism, this parameter occurs in eq. S4 and determines how quickly an individual's probability of dying increases (via the hazard rate). It is not

straightforward to relate this parameter to a process in reality. Thus, we chose to scale it with temperature based on its unit (i.e., includes time), while following the reasoning described in the main script, assuming that temperature acts by stretching or compressing the time axis.

Model equations and parameters

Table S1: Model equations and symbols for the parameters and variables used. *Symbols used are given with their explanation and unit of the parameter and variables they represent. Model parameters estimated (and corrected for temperature) in the GUTS application are listed for toxicokinetics and toxicodynamics. SD = stochastic death; IT = individual tolerance*

Model	Equation	
Arrhenius	$k_x(T) = k_{x,T_{ref}} \cdot e^{\left(\frac{T_A}{T_{ref}} - \frac{T_A}{T}\right)}$<br>$k_x$ is a rate parameter: k_u, k_e, k_m, k_{em}, k_r, h_b or b_i<br>$k_{x,ref}$ is the respective rate parameter at the reference temperature	eq. (S1)
Toxicokinetics	For flupyradifurone:<br>$\frac{dC_i(t)}{dt} = k_u \cdot C_w(t) - k_e \cdot C_i(t)$	eq. (S2a)
	For imidacloprid and imidacloprid-olefin<br>$\frac{dC_i(t)}{dt} = (k_u \cdot C_w(t) - k_e \cdot C_i(t)) - k_m \cdot C_i(t)$	eq. (S2b)
	$\frac{dC_m(t)}{dt} = k_m \cdot C_i(t) - k_{em} \cdot C_m(t)$	eq. (S2c)
Damage dynamics	$\frac{dD_i(t)}{dt} = k_r \cdot (C_i(t) - D_i(t))$	eq. (S3)
SD model	$h_z = b_i \cdot \max(0, D_i(t) - m_i) + h_b \;\; with$	eq. (S4)
	$\frac{S_{SD}(t)}{dt} = -h_z \cdot S$	eq. (S5)
IT model	$F(t) = \frac{1}{1 + \left(\frac{D_{i,max}}{m_i}\right)^{\beta}}$	eq. (S6)
	$D_{i,max} = \max_{0<\tau<t} D_i(\tau)$	eq. (S7)
	$\beta = \frac{\log 39}{\log F_s}$	eq. (S8)
	$S_{IT}(t) = (1 - F(D_{i,max})) \cdot e^{-h_b \cdot t}$	eq. (S9)

Symbol	Explanation	Unit
Temperature parameters		
T	Temperature	K
T_{ref}	Reference temperature (20 °C = 293.15 K)	K
T_A-tk	Arrhenius temperature used to correct TK parameter	K
T_A-td	Arrhenius temperature used to correct TD parameter	K
GUTS model parameter and variables		
C_i	Concentration of the chemical inside the organism	$\mu g \cdot kg^{-1}$
C_w	Concentration of the chemical in the exposure medium	$\mu g \cdot L^{-1}$

C_m	Concentration of the metabolite inside the organism	$\mu g \cdot L^{-1}$
D_i	Scaled damage, referenced to internal concentration	$\mu g \cdot kg^{-1}$
h_z	Hazard rate for an individual with threshold z	$[d^{-1}]$
S	Survival probability in a population of individuals	[-]
$F(m_i, \beta)$	Cumulative log-logistic distribution function of the thresholds	[-]
β	Shape parameter for the distribution of thresholds (F)	[-]
t	Time	day
Toxicokinetic parameter estimated in the TK applications		
k_u or $k_u(T)$	Uptake rate of chemicals into the organism (corrected for temperature, at 20 °C)	$L \cdot kg^{-1} \cdot day^{-1}$
k_e or $k_e(T)$	Elimination rate of the chemicals from the organism (corrected for temperature, at 20 °C)	d^{-1}
k_m or $k_m(T)$	Formation rate of the metabolite within the organism (corrected for temperature, at 20 °C)	$L \cdot kg^{-1} \cdot day^{-1}$
k_{em} or $k_{em}(T)$	Elimination rate of the metabolite from the organism (corrected for temperature, at 20 °C)	d^{-1}
Toxicodynamic parameter estimated in the GUTS applications		
k_r or $k_r(T)$	Damage repair rate corrected for temperature (corrected for temperature, at 20 °C)	d^{-1}
h_b or $h_b(T)$	Background hazard rate (corrected for temperature, at 20 °C)	d^{-1}
m_i	Median of the distribution of thresholds (F), referenced to internal concentration	$\mu g \cdot kg^{-1}$
b_i or $b_i(T)$	Killing rate, referenced to internal concentration(corrected for temperature, at 20 °C) – For SD model only	$kg \cdot \mu g^{-1} \cdot d^{-1}$
F_s	Fraction spread in the distribution of thresholds (F) – For IT model only	[-]

Toxicokinetic modeling

Table S2: Temperature corrected toxicokinetic model parameter (TK-T) for imidacloprid and flupyradifurone in Gammarus pulex. *The parameters values and the kinetic bioconcentration factor (BCFkin) are listed with their upper and lower 95 % confidence intervals (CI) and were estimated for a reference temperature (T_{ref}) set to 20 °C.*

Parameter	Unit	Imidacloprid		Flupyradifurone	
$k_u(T)$ or k_u	$L \cdot kg^{-1} \cdot day^{-1}$	3.250	(3.077 - 3.446)	1.313	(1.229 - 1.416)
$k_e(T)$	d^{-1}	0.140	(0.089 - 0.155)	0.135	(0.118 - 0.158)
$k_m(T)$	$L \cdot kg^{-1} \cdot day^{-1}$	0.022	(0.014 - 0.068)	-	-
$k_{em}(T)$	d^{-1}	0.488	(0.219 - 1.542)	-	-
T_A-tk	K	3044	(2316 - 3724)	9243	(1943 - 15870)
BCF_{kin}	[-]	20.14	(19.46 - 20.76)	9.70	(8.76 - 10.57)

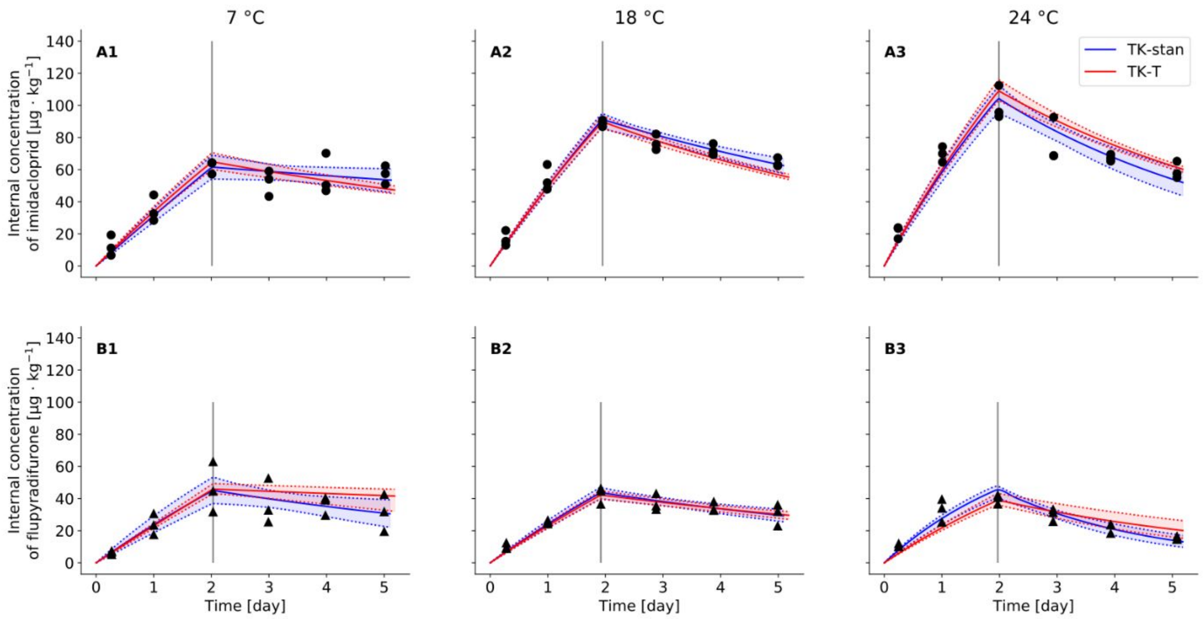

Figure S1: Internal concentration of neonicotinoids in Gammarus pulex at different temperatures. *Black symbols are measured internal concentrations at 7, 18, and 24°C, respectively. Blue solid lines are the TK standard model fits (temperature considered implicitly, from [7], and red solid lines are TK-T model fits (temperature considered explicitly) with lower and upper confidence intervals (dotted lines). The vertical grey line marks the transition timepoint from uptake to elimination phase for each temperature and chemical. Upper panel A1-A3: imidacloprid; lower panel B1-B3: flupyradifurone.*

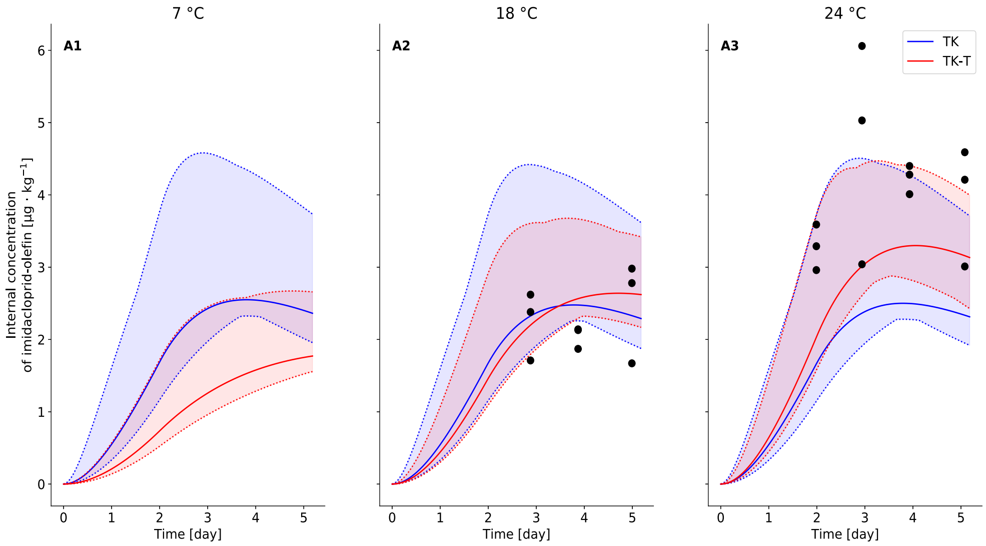

Figure S2: Internal concentration of imidacloprid-olefin in Gammarus pulex at different temperatures. *Black dots are measured internal concentrations at 7 (A1), 18 (A2), and 24 °C (A3), respectively. Blue solid lines are the TK model fits (without temperature correction), and red solid lines are TK-T model fits (with temperature correction) with lower and upper confidence intervals (dotted lines).*

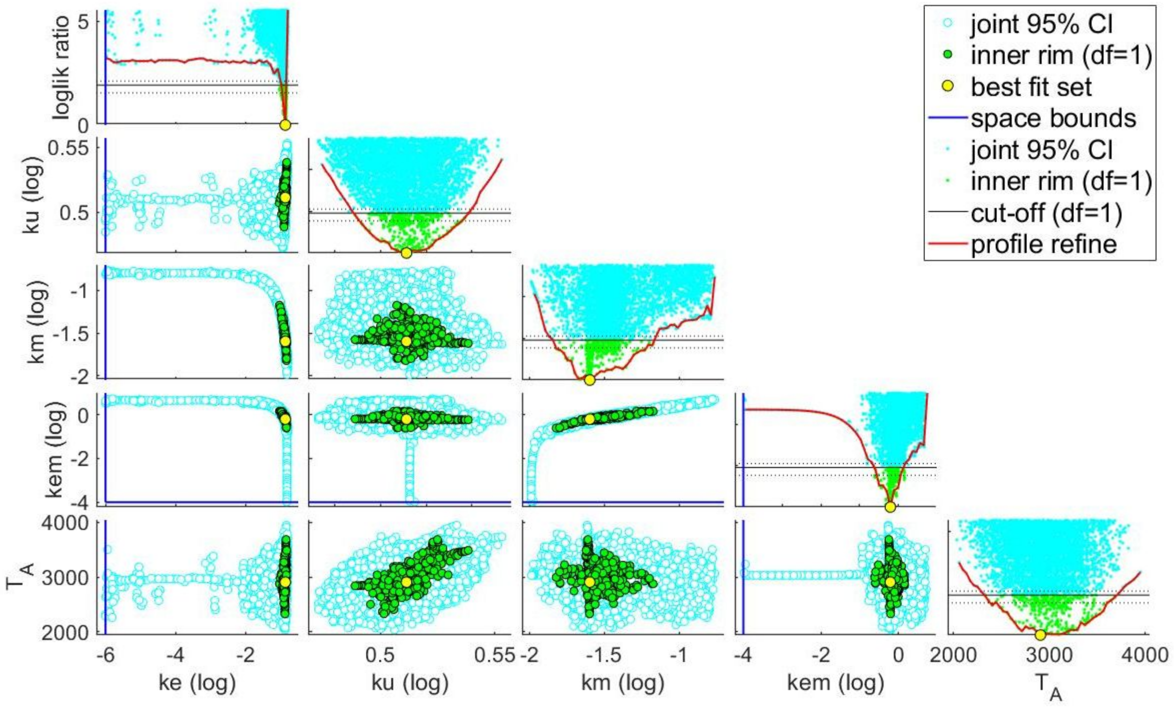

Figure S3: Parameter-space plot for the fit of TK-T to internal concentration measurements of imidacloprid and its metabolite imidacloprid-olefin in Gammarus pulex. *The plots on the diagonal show the profile likelihoods for the individual parameters and the other plots are the 95% joint confidence regions. Yellow dots mark the best-fit values, green dots show parameter sets within the critical value (horizontal black line). The parameter sets between the dotted horizontal lines are used for the confidence intervals on model curves. The parameter symbols and their units correspond to Table S1.*

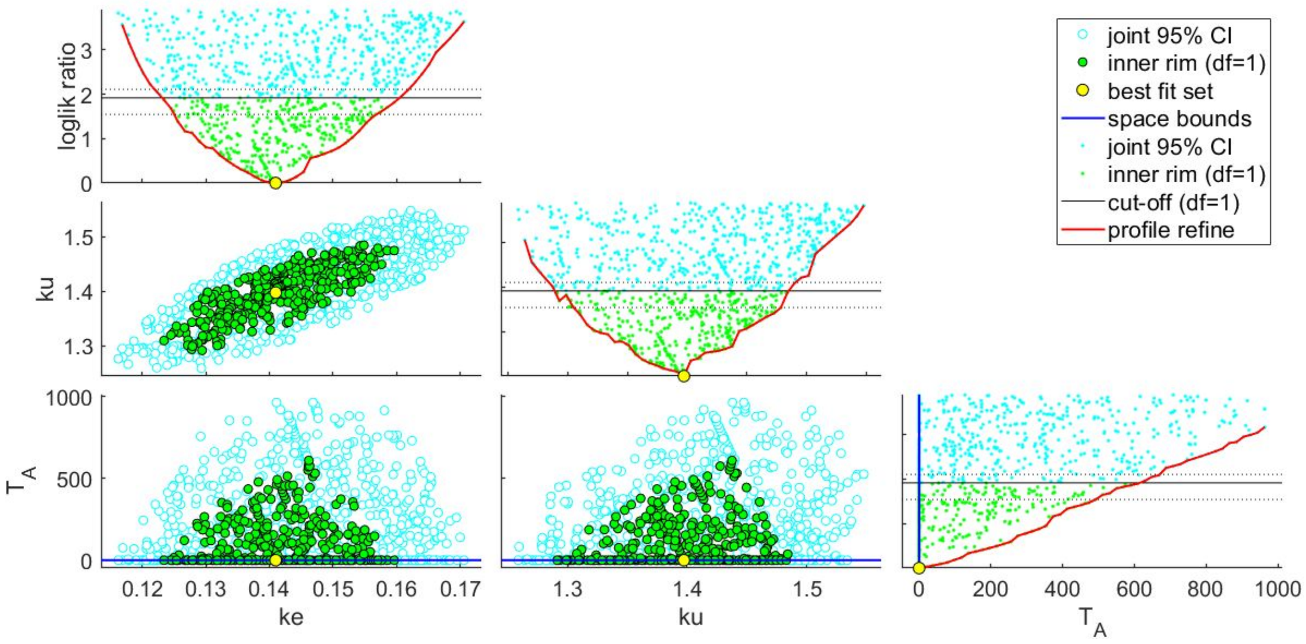

Figure S4: Parameter-space plot for the fit of TK-T to internal concentration measurements of flupyradifurone in Gammarus pulex, when T_A corrects both rates. *Yellow dots mark the best-fit values, green dots show parameter sets within the critical value (horizontal black line). The parameter sets between the dotted horizontal lines are used for the confidence intervals on model curves. The parameter symbols and their units correspond to Table S1. Note: T_A can not be distinguished from zero when correcting k_u.*

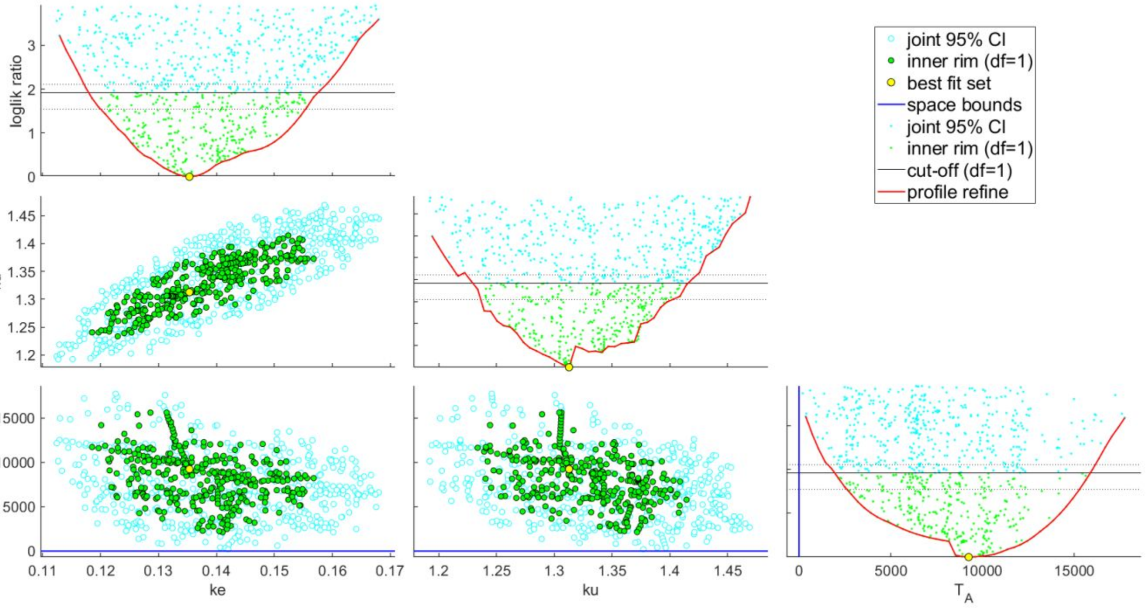

Figure S5: Parameter-space plot for the fit of TK-T to internal concentration measurements of flupyradifurone in Gammarus pulex, when T_A corrects only k_e. *Yellow dots mark the best-fit values, green dots show parameter sets within the critical value (horizontal black line). The parameter sets between the dotted horizontal lines are used for the confidence intervals on model curves. The parameter symbols and their units correspond to Table S1.*

Effect modeling (GUTS-FULL-T)

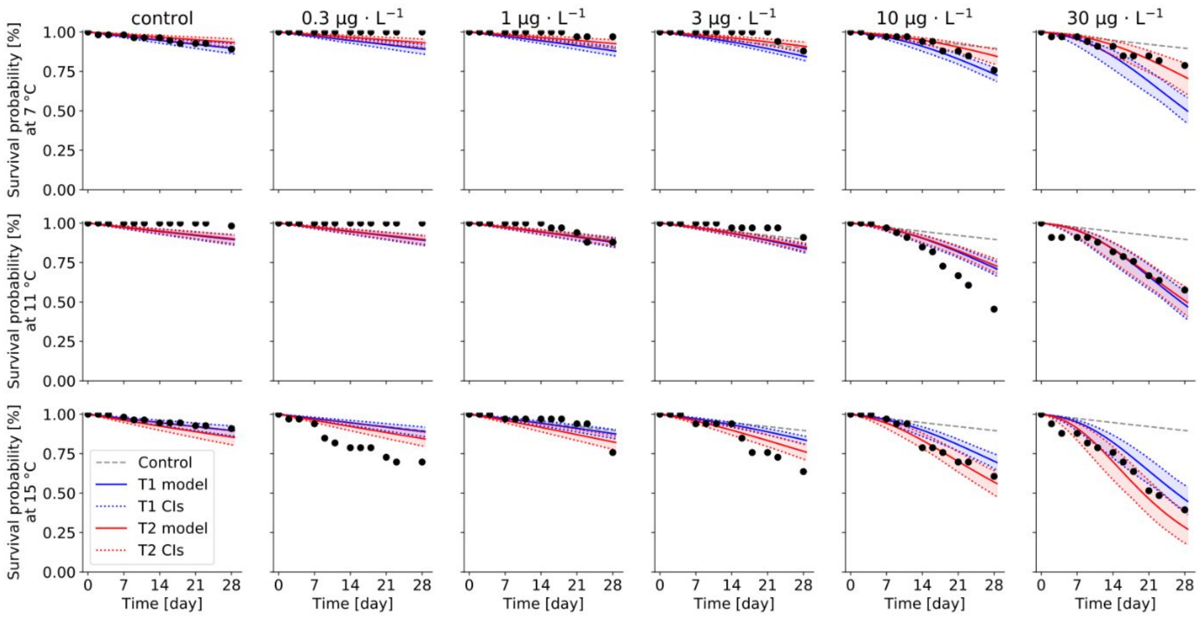

Figure S6: Concentration-response curves (SD models) for the survival of Gammarus pulex exposed to imidacloprid at different temperatures. *Black dots represent the measured survival (replicates pooled) at the different exposure levels in µg per L (column headings) and the different temperatures in degrees Celsius (y-axis label). The blue line shows the GUTS-FULL-T1-SD model (i.e., only toxicokinetic parameters corrected for temperature) prediction for survival and the dotted blue lines the boundaries of its 95 % confidence interval (blue area). The red line shows the GUTS-FULL-T2-SD model (i.e., also toxicodynamic parameters corrected for temperature) prediction for survival and the dotted red lines the boundaries of its 95 % confidence interval (red area). The gray dotted line shows the control mortality as modeled under GUTS-FULL-T1-SD for each temperature.*

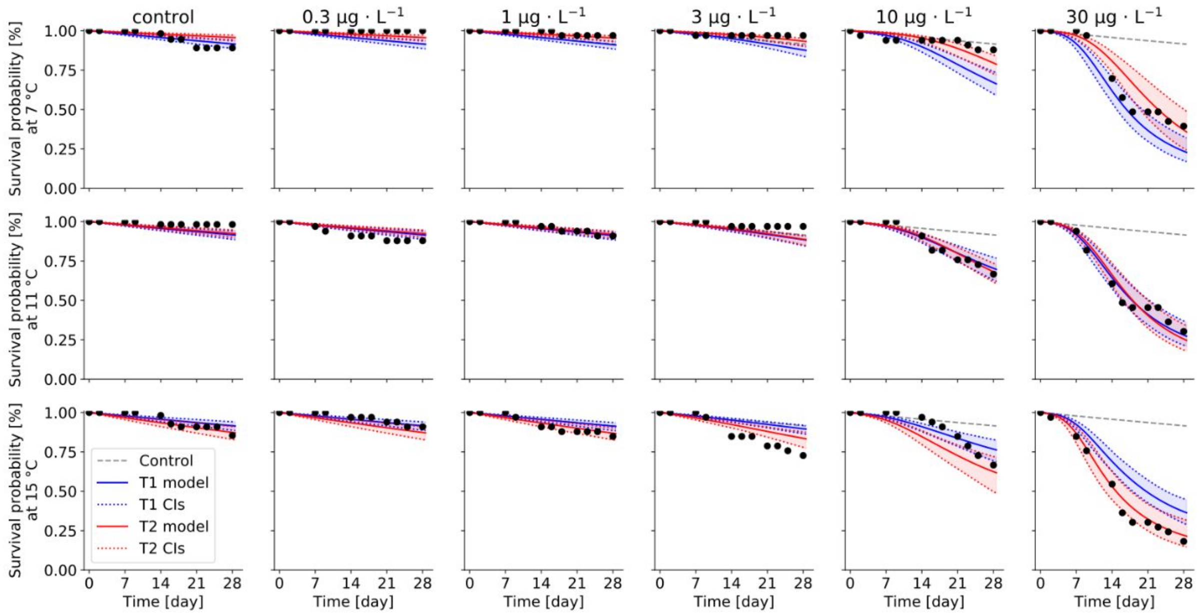

Figure S7: Concentration-response curves (IT models) for the survival of Gammarus pulex exposed to flupyradifurone at different temperatures. *Black dots represent the measured survival (replicates pooled) at the different exposure levels in µg per L (column headings) and the different temperatures in degrees Celsius (y-axis label). The blue line shows the GUTS-FULL-T1-IT model (i.e., only toxicokinetic parameters corrected for temperature) prediction for survival and the dotted blue lines the boundaries of its 95 % confidence interval (blue area). The red line shows the GUTS-FULL-T2-IT model (i.e., also toxicodynamic parameters corrected for temperature) prediction for survival and the dotted red lines the boundaries of its 95 % confidence interval (red area). The gray dotted line shows the control mortality as modeled under GUTS-FULL-T1-SD for each temperature.*

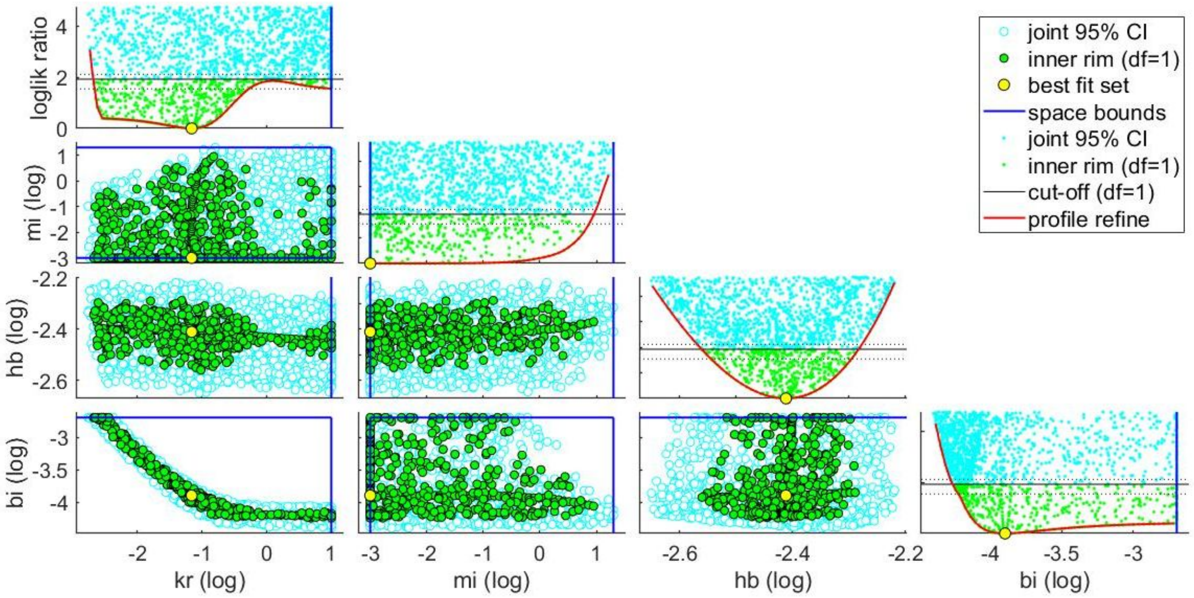

Figure S8:. Parameter-space plot for fit of GUTS-FULL-T1-SD model parameter for imidacloprid. *The plots on the diagonal show the profile likelihoods for the individual parameters and the other plots are the 95% joint confidence regions. Yellow dots mark the best-fit values, green dots show parameter sets within the critical value (horizontal black line). The parameter sets between the dotted horizontal lines are used for the confidence intervals on model curves. The parameter symbols and their units correspond to Table S1.*

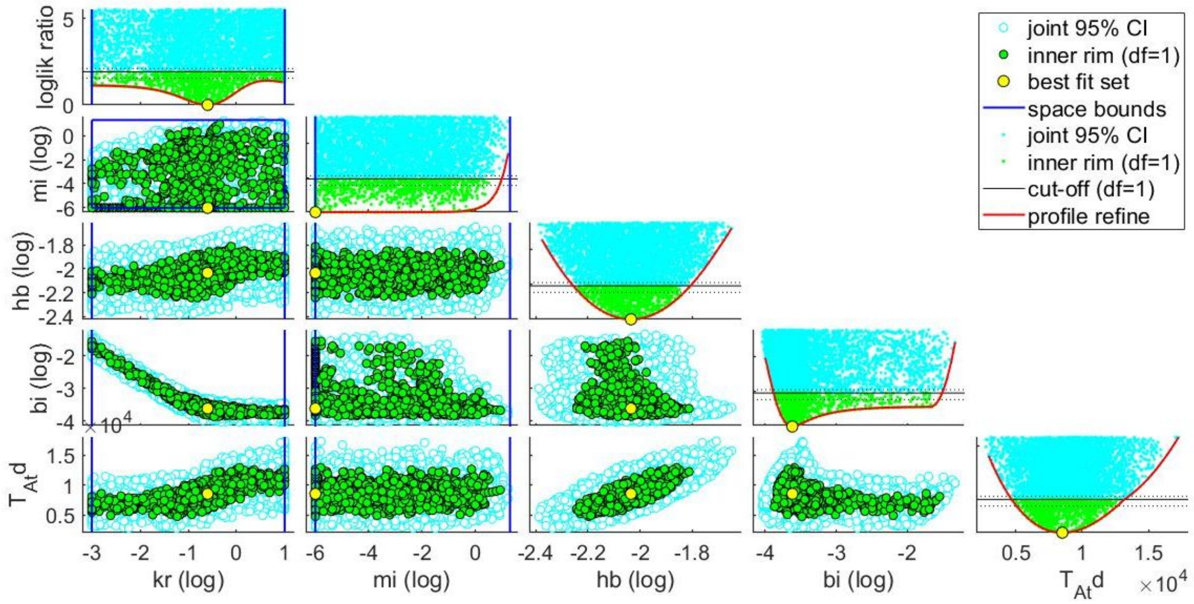

Figure S9: Parameter-space plot for fit of GUTS-FULL-T2-SD model parameter for imidacloprid. *The plots on the diagonal show the profile likelihoods for the individual parameters and the other plots are the 95% joint confidence regions. Yellow dots mark the best-fit values, green dots show parameter sets within the critical value (horizontal black line). The parameter sets between the dotted horizontal lines are used for the confidence intervals on model curves. The parameter symbols and their units correspond to Table S1.*

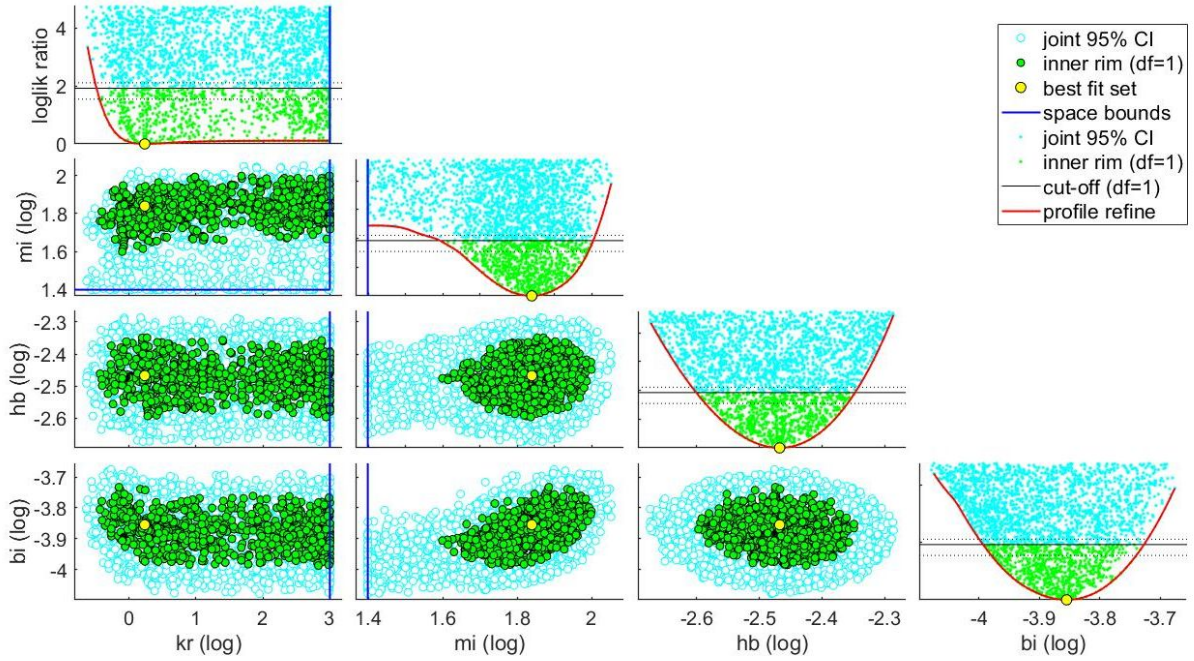

Figure S10: Parameter-space plot for fit of GUTS-FULL-T1-SD model parameter for flupyradifurone. *The plots on the diagonal show the profile likelihoods for the individual parameters and the other plots are the 95% joint confidence regions. Yellow dots mark the best-fit values, green dots show parameter sets within the critical value (horizontal black line). The parameter sets between the dotted horizontal lines are used for the confidence intervals on model curves. The parameter symbols and their units correspond to Table S1.*

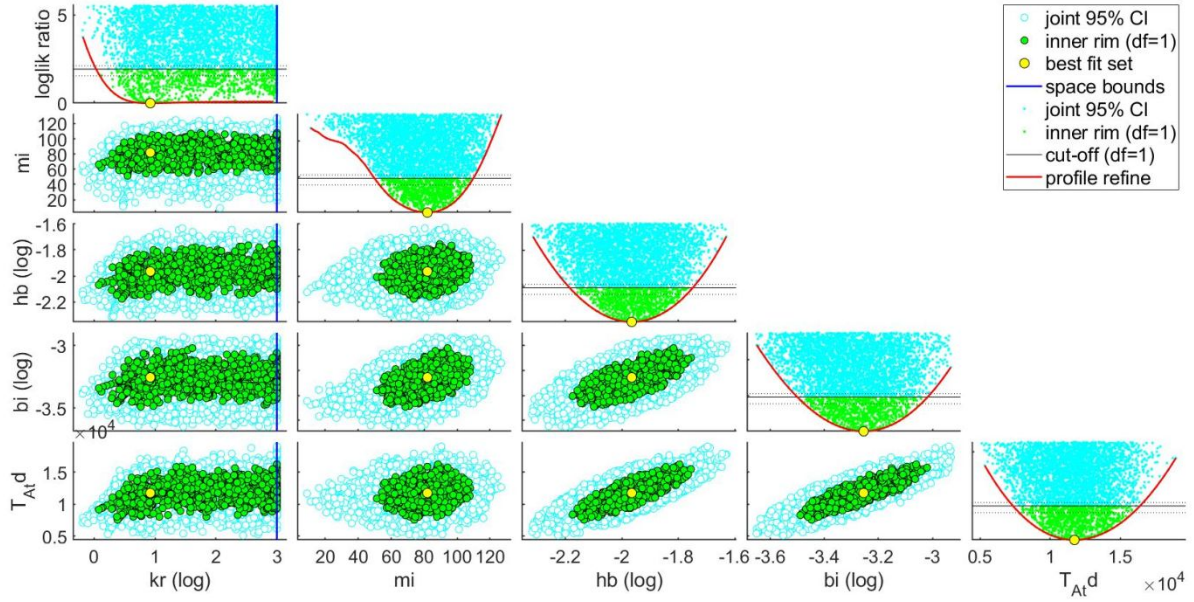

Figure S11: Parameter-space plot for fit of GUTS-FULL-T2-SD model parameter for flupyradifurone. *The plots on the diagonal show the profile likelihoods for the individual parameters and the other plots are the 95% joint confidence regions. Yellow dots mark the best-fit values, green dots show parameter sets within the critical value (horizontal black line). The parameter sets between the dotted horizontal lines are used for the confidence intervals on model curves and LCx values. The parameter symbols and their units correspond to Table S1.*

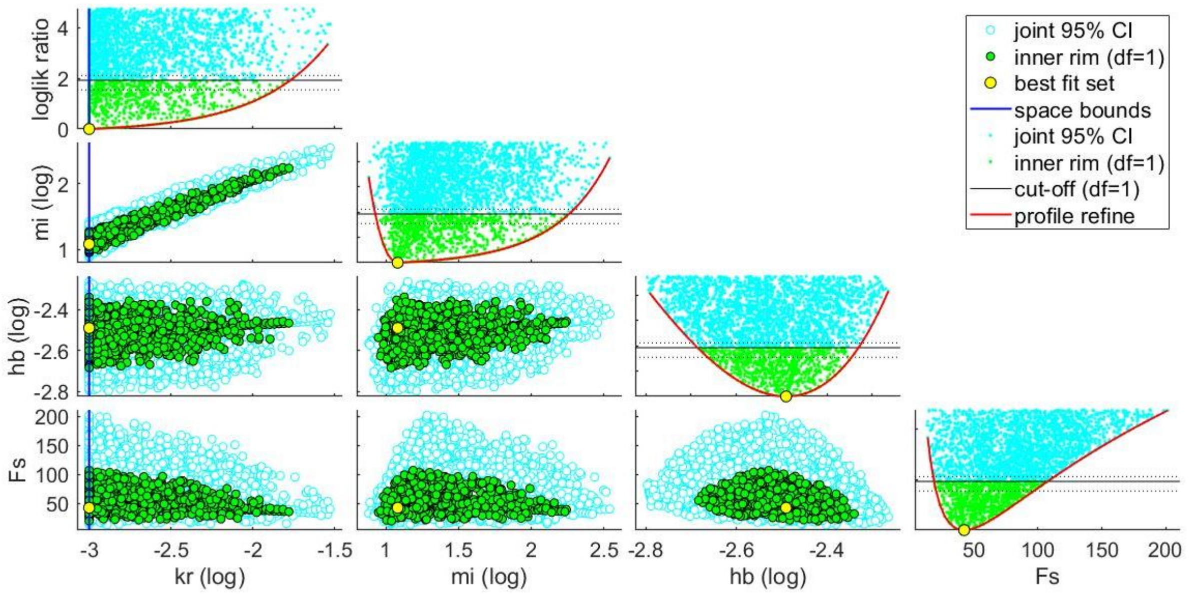

Figure S12: Parameter-space plot for fit of GUTS-FULL-T1-IT model parameter for imidacloprid. *The plots on the diagonal show the profile likelihoods for the individual parameters and the other plots are the 95% joint confidence regions. Yellow dots mark the best-fit values, green dots show parameter sets within the critical value (horizontal black line). The parameter sets between the dotted horizontal lines are used for the confidence intervals on model curves. The parameter symbols and their units correspond to Table S1.*

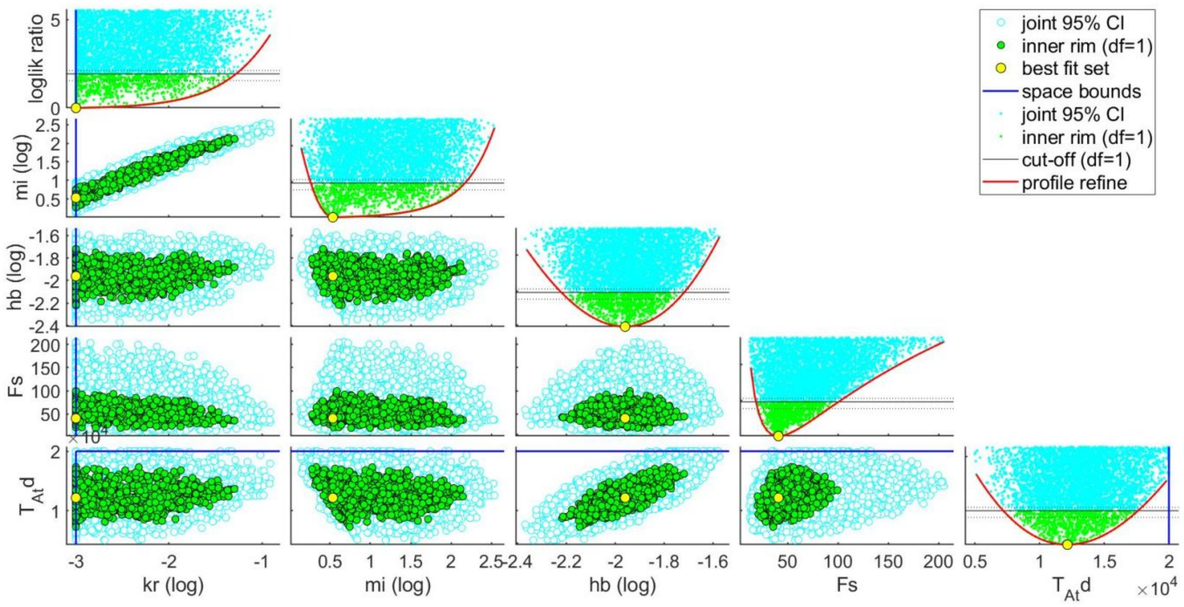

Figure S13: Parameter-space plot for fit of GUTS-FULL-T2-IT model parameter for imidacloprid. *The plots on the diagonal show the profile likelihoods for the individual parameters and the other plots are the 95% joint confidence regions. Yellow dots mark the best-fit values, green dots show parameter sets within the critical value (horizontal black line). The parameter sets between the dotted horizontal lines are used for the confidence intervals on model curves and LCx values. The parameter symbols and their units correspond to Table S1.*

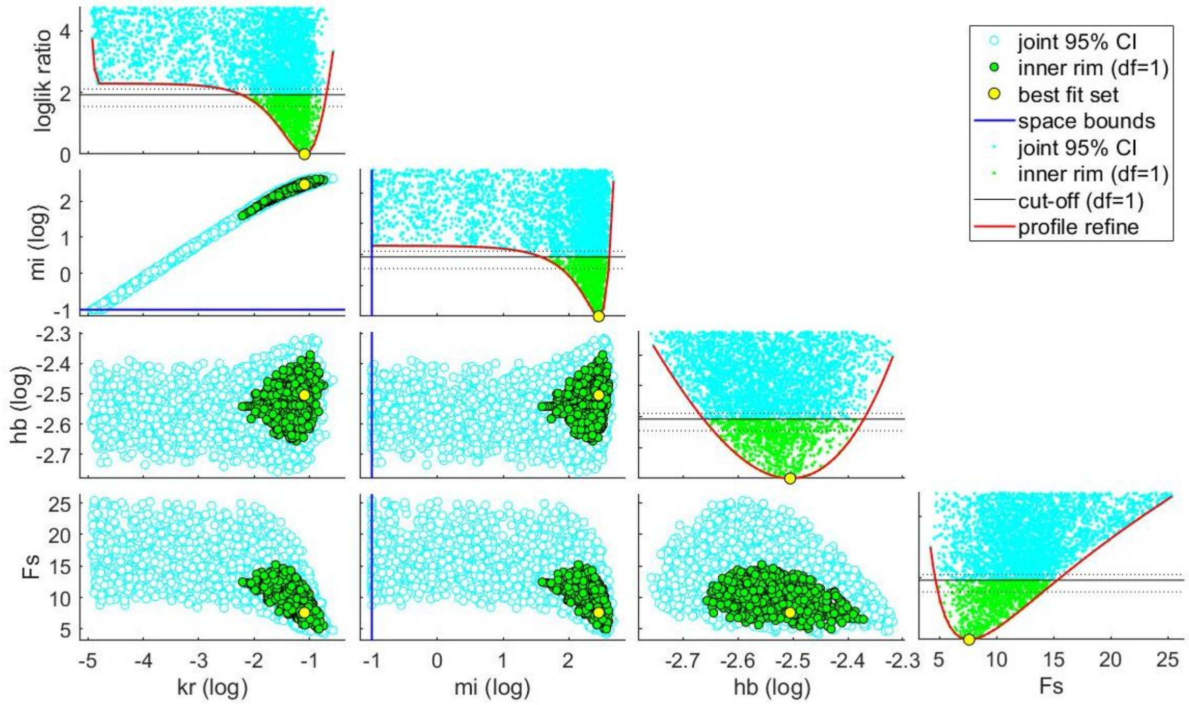

Figure S14: Parameter-space plot for fit of GUTS-FULL-T1-IT model parameter for flupyradifurone. *The plots on the diagonal show the profile likelihoods for the individual parameters and the other plots are the 95% joint confidence regions. Yellow dots mark the best-fit values, green dots show parameter sets within the critical value (horizontal black line). The parameter sets between the dotted horizontal lines are used for the confidence intervals on model curves. The parameter symbols and their units correspond to Table S1.*

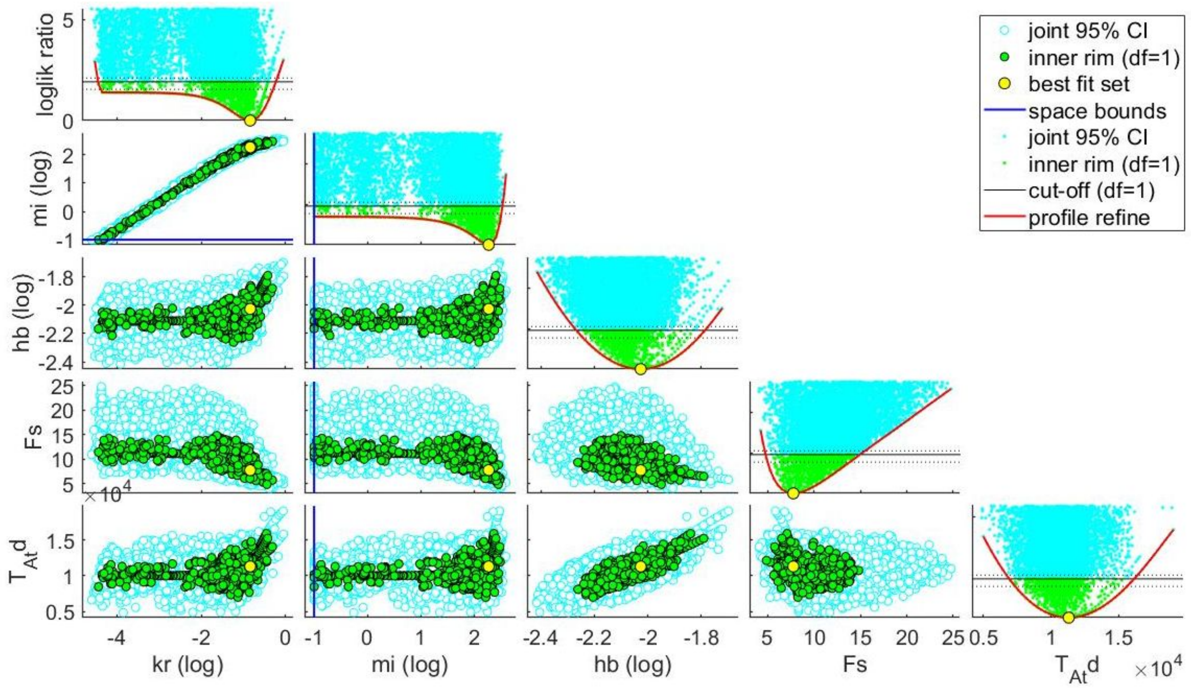

Figure S15: Parameter-space plot for fit of GUTS-FULL-T2-IT model parameter for flupyradifurone. *The plots on the diagonal show the profile likelihoods for the individual parameters and the other plots are the 95% joint confidence regions. Yellow dots mark the best-fit values, green dots show parameter sets within the critical value (horizontal black line). The parameter sets between the dotted horizontal lines are used for the confidence intervals on model curves. The parameter symbols and their units correspond to Table S1.*

References

(1) Felten, V.; Charmantier, G.; Mons, R.; Geffard, A.; Rousselle, P.; Coquery, M.; Garric, J.; Geffard, O. Physiological and Behavioural Responses of Gammarus Pulex (Crustacea: Amphipoda) Exposed to Cadmium. *Aquatic Toxicology* **2008**, *86* (3), 413–425. https://doi.org/10.1016/j.aquatox.2007.12.002.

(2) Molecular Diffusion. *Wikipedia*; 2021.

(3) Camp, A. A.; Buchwalter, D. B. Can't Take the Heat: Temperature-Enhanced Toxicity in the Mayfly Isonychia Bicolor Exposed to the Neonicotinoid Insecticide Imidacloprid. *Aquatic Toxicology* **2016**, *178*, 49–57. https://doi.org/10.1016/j.aquatox.2016.07.011.

(4) Sutcliffe, D. W. Quantitative Aspects of Oxygen Uptake by Gammarus (Crustacea, Amphipoda): A Critical Review. *Freshwater Biology* **1984**, *14* (5), 443–489. https://doi.org/10.1111/j.1365-2427.1984.tb00168.x.

(5) Buchwalter, D. B.; Jenkins, J. J.; Curtis, L. R. Temperature Influences on Water Permeability and Chlorpyrifos Uptake in Aquatic Insects with Differing Respiratory Strategies. *Environmental Toxicology and Chemistry* **2003**, *22* (11), 2806–2812. https://doi.org/10.1897/02-350.

(6) Huang, A.; Mangold-Döring, A.; Guan, H.; Boerwinkel, M.-C.; Belgers, D.; Focks, A.; van den Brink, P. Data for: The Effect of Temperature on Toxicokinetics and the Chronic Toxicity of Insecticides towards Gammarus Pulex. **2022**, *2*. https://doi.org/10.17632/6dbgkhzxvx.2.

(7) Huang, A.; Mangold-Döring, A.; Guan, H.; Boerwinkel, M.-C.; Belgers, D.; Focks, A.; Brink, P. J. V. den. The Effect of Temperature on Toxicokinetics and the Chronic Toxicity of Insecticides towards Gammarus Pulex. *accepted manuscript at Science of the Total Environment* **2022**.

(8) Casida, J. E. Neonicotinoid Metabolism: Compounds, Substituents, Pathways, Enzymes, Organisms, and Relevance. *J. Agric. Food Chem.* **2011**, *59* (7), 2923–2931. https://doi.org/10.1021/jf102438c.

(9) Honda, H.; Tomizawa, M.; Casida, J. E. Neonicotinoid Metabolic Activation and Inactivation Established with Coupled Nicotinic Receptor-CYP3A4 and -Aldehyde Oxidase Systems. *Toxicology Letters* **2006**, *161* (2), 108–114. https://doi.org/10.1016/j.toxlet.2005.08.004.

(10) Jager, T.; Ashauer, R. *Modelling Survival under Chemical Stress A Comprehensive Guide to the GUTS Framework*; 2018.

(11) *Mode of Action Classification | Insecticide Resistance Management*. IRAC. https://irac-online.org/mode-of-action/classification-online/ (accessed 2022-08-15).

(12) Tomizawa, M.; Casida, J. E. NEONICOTINOID INSECTICIDE TOXICOLOGY: Mechanisms of Selective Action. *Annu. Rev. Pharmacol. Toxicol.* **2004**, *45* (1), 247–268. https://doi.org/10.1146/annurev.pharmtox.45.120403.095930.

(13) Maloney, E. M. Cumulative Toxicities of Neonicotinoid Insecticides and Their Mixtures to Sensitive Freshwater Insects. Ph.D. Dissertation, University of Saskatchewan, Saskatoon, SK, Canada, 2020.

(14) Jones, A. K.; Sattelle, D. B. Diversity of Insect Nicotinic Acetylcholine Receptor Subunits. In *Insect Nicotinic Acetylcholine Receptors*; Thany, S. H., Ed.; Advances in Experimental Medicine and Biology; Springer: New York, NY, 2010; pp 25–43. https://doi.org/10.1007/978-1-4419-6445-8_3.

(15) Casida, J. E.; Durkin, K. A. Neuroactive Insecticides: Targets, Selectivity, Resistance, and Secondary Effects. *Annu. Rev. Entomol.* **2013**, *58* (1), 99–117. https://doi.org/10.1146/annurev-ento-120811-153645.

(16) Gupta, R. C. *Veterinary Toxicology: Basic and Clinical Principles*; Academic Press, 2012.

(17) Fayuk, D.; Yakel, J. L. Regulation of Nicotinic Acetylcholine Receptor Channel Function by Acetylcholinesterase Inhibitors in Rat Hippocampal CA1 Interneurons. *Mol Pharmacol* **2004**, *66* (3), 658–666. https://doi.org/10.1124/mol.104.000042.

(18) Thany, S. H. Agonist Actions of Clothianidin on Synaptic and Extrasynaptic Nicotinic Acetylcholine Receptors Expressed on Cockroach Sixth Abdominal Ganglion. *NeuroToxicology* **2009**, *30* (6), 1045–1052. https://doi.org/10.1016/j.neuro.2009.06.013.

(19) Oliveira, E. E.; Schleicher, S.; Büschges, A.; Schmidt, J.; Kloppenburg, P.; Salgado, V. L. Desensitization of Nicotinic Acetylcholine Receptors in Central Nervous System Neurons of the Stick Insect (Carausius Morosus) by Imidacloprid and Sulfoximine Insecticides. *Insect Biochemistry and Molecular Biology* **2011**, *41* (11), 872–880. https://doi.org/10.1016/j.ibmb.2011.08.001.
